# Organ-Specific Distribution of Antimycobacterial Neolignans in *Piper rivinoides* and UHPLC-HRMS/MS Analysis of Its Extracts

**DOI:** 10.3390/molecules30244682

**Published:** 2025-12-06

**Authors:** Jéssica Sales Felisberto, Thayssa Ferreira Fagundes, Lorraynne Oliveira-Souza, Bruno Henrique Gomes de Souza, Daniel Machado de Brito, Jeferson Adriano Assunção, Samik Lourenço Massau, Marlon H. de Araújo, Michelle Frazão Muzitano, Sanderson Dias Calixto, Thatiana Lopes Biá Ventura Simão, Andre Mesquita Marques, Ygor Jessé Ramos, Davyson de Lima Moreira

**Affiliations:** 1 Postgraduate Program in Plant Biology, Institute of Biology Roberto Alcantara Gomes, State University of Rio de Janeiro, São Francisco Xavier, Rio de Janeiro 20950-000, RJ, Brazil; jessicka.salles@gmail.com (J.S.F.); lorraynneoliveira282@gmail.com (L.O.-S.); bhgomesdesouza@gmail.com (B.H.G.d.S.); danielmachado.cedae@gmail.com (D.M.d.B.); 2 Botanical Garden Research Institute of Rio de Janeiro, Rua Pacheco Leão, 915, Jardim Botânico, Rio de Janeiro 22460-030, RJ, Brazil; thayssasffagundes@gmail.com (T.F.F.); jefersonadriano.sa@gmail.com (J.A.A.); samiklourecomassau@hotmail.com (S.L.M.); 3 Recenor Biology Laboratory, Center of Biosciences and Biotechnology, State University of North Fluminense Darcy Ribeiro, Rua Alberto Lamego, 2000, Campos dos Goytacazes 28013-602, RJ, Brazil; sandersoncalixto@yahoo.com.br (S.D.C.); thativentura@uenf.com (T.L.B.V.S.); 4 Laboratory of Bioatives Products, Institute of Pharmaceutical Sciences, Federal University of Rio de Janeiro, Rua Alcides da Conceição, 159, Macaé 27933-378, RJ, Brazil; marlon.heggdorne@gmail.com (M.H.d.A.); mfmuzitano@macae.ufrj.br (M.F.M.); 5 Laboratory of Technology for Biodiversity in Health/TecBio, Farmanguinhos, Oswaldo Cruz Foundation, Rua Sizenando Nabuco, 100, Manguinhos, Rio de Janeiro 21041-250, RJ, Brazil; andrefarmaciarj@yahoo.com.br; 6 Farmácia da Terra Laboratory, Faculty of Pharmacy, Federal University of Bahia, Rua Barão de Jeremoabo, 147, Ondina, Salvador 40170-115, BA, Brazil; 7 Postgraduate Program in Translational Research in Drugs and Medicines, Pharmaceutical Technology Institute, Farmanguinhos, Fiocruz, Rua Sizenando Nabuco, 100, Manguinhos, Rio de Janeiro 21041-250, RJ, Brazil

**Keywords:** Piperaceae, antimycobacterials, LC-MS/MS, LC-DAD-UV, atlantic forest

## Abstract

This multidisciplinary study investigates *Piper rivinoides*, a Brazilian medicinal species, focusing on its chemical composition and antimycobacterial potential. UHPLC-HRMS/MS of leaves, stems, branches, and roots revealed 58 compounds, including neolignans, lignanamides, triterpenes, flavonoids, and carotenoids. Fourteen metabolites, notably benzofuran neolignans and pentacyclic triterpenes are annotated here for the first time. Quantitative analyses by HPLC-DAD-UV showed that eupomatenoid-5, eupomatenoid-6, and conocarpan were most abundant in leaves, reaching amounts approximately twice those found in branches and stems and about ten times higher than in roots, supporting the optimal defense theory and organ-specific accumulation of bioactive metabolites. Biological assays against *Mycobacterium tuberculosis* strains H37Rv and M299 revealed strong inhibitory activity for the leaf extract and isolated neolignans. Eupomatenoid-5 and eupomatenoid-6 achieved inhibition comparable to rifampicin, with low MIC_50_ values, while conocarpan exhibited moderate activity. Antimycobacterial effects were more pronounced against the H37Rv strain, although relevant inhibition was also observed for the hypervirulent M299 strain. These findings highlight *P. rivinoides* as a rich source of benzofuran neolignans and promising antimycobacterial properties. The integration of advanced mass spectrometric analyses with bioassays demonstrates the value of combining chemical and biological approaches to uncover novel natural products. The putative identification of new neolignans and triterpenes, along with the confirmation of potent antimycobacterial activity, provides a robust foundation for further studies on biosynthesis, structure–activity relationships, and potential biotechnological applications.

## 1. Introduction

Tuberculosis (TB), a chronic granulomatous infection caused by members of the *Mycobacterium tuberculosis* complex, remains one of the leading causes of death from infectious diseases worldwide. According to the 2024 Global Tuberculosis Report from the World Health Organization (WHO), approximately 10.8 million people developed TB globally in 2023. Although the number of deaths declined compared to the previous two years, TB has once again become the leading cause of death from a single infectious agent [1].

*M. tuberculosis* infection manifests in two forms: an active, where the bacterium multiplies in the lungs causing symptoms and enabling transmission; and a latent form, in which the pathogen remains dormant without clinical signs or transmissibility but may reactivate later in the life cycle. A central feature of TB pathogenesis is the ability of *M. tuberculosis* to persist intracellularly within diverse populations of myeloid cells. The progression of infection is modulated by host genetic factors, comorbidities, environmental influences, and microbial virulence determinants, many of which remain incompletely understood. To establish infection, *M. tuberculosis* evades macrophage- and neutrophil-mediated defenses by blocking phagolysosomal trafficking, allowing intracellular survival. The bacterium infects both embryonically derived alveolar macrophages and various phenotypically distinct macrophage populations of hematopoietic origin [2,3,4]. Clinically, TB presents a broad spectrum—from localized pulmonary cavitary lesions, the most common and transmissible form, to focal or disseminated infections affecting multiple organ systems. Despite their severity, disseminated forms are less advantageous for the bacterium in terms of transmission potential [4].

Despite the availability of first-line antimycobacterial drugs such as rifampicin and isoniazid, as well as the Bacillus Calmette–Guérin (BCG) vaccine for prevention, the prolonged duration of treatment and the emergence of multidrug-resistant *M. tuberculosis* (MDR-TB) strains present significant challenges for conventional therapeutic approaches [5,6]. Consequently, the development of new strategies and the discovery of novel bioactive compounds to combat TB are considered critical priorities in the field of infectious disease research.

Given the urgent need for novel antimycobacterial therapies, secondary metabolites from natural sources have attracted increasing interest due to their structural diversity and potential to overcome drug resistance [7]. Examples include flavonoids such as orientin, isovitexin, and apigenin, which have shown inhibitory activity against *M. tuberculosis* H37Rv, as well as coumarins and terpenoids (e.g., protopanaxatriol) recognized for their antimycobacterial potential [8]. These compounds offer unique mechanisms of action and have historically contributed significantly to antimicrobial drug discovery, reinforcing their relevance in the search for new treatments against *M. tuberculosis* [7,8].

Species of the genus *Piper* L. (Piperaceae), such as *P. aduncum* L., *P. betle* L., and *P. multinodum* C. DC., have emerged as promising sources of compounds with antimycobacterial potential, as demonstrated by various biological assays [9,10,11]. *Piper rivinoides* Kunth, a native species to Brazil and widely used in traditional medicine to treat wounds, ulcers, vaginal discharge, bleeding, and even oral problems, with leaves commonly prepared as decoctions or hydroalcoholic tinctures. This plant exhibits a specialized metabolism rich in terpenoids (monoterpenes and sesquiterpenes) and neolignans [12,13,14], with reported fungicidal, antiparasitic, antimicrobial, antinociceptive, and cytotoxic activities [13,15,16,17]. However, detailed ethnobotanical information, such as the harvesting period for medicinal purposes, is not available in the literature.

The production of these specialized metabolites is influenced by spatiotemporal factors, reflecting chemical phenotypic plasticity [18,19,20], which leads to qualitative and quantitative variations among plant organs and may impact on their bioactivity [21]. Given that the pharmacological activity of these metabolites may vary depending on their tissue-specific localization. Then, analysis of the chemical composition and antimycobacterial activity of extracts from leaves, branches, stems, and roots of *P. rivinoides* is essential to identify vegetal parts with the greatest therapeutic potential and to elucidate the organ-specific distribution of pharmacologically relevant compounds [18,19,20,21,22,23].

The present study focuses on the metabolic putatively annotated or quantified with isolated neolignans, as well as bioactivity evaluation of extracts from the leaves, stems, branches, and roots of *P. rivinoides* cultivated in Brazil. Ultra-high-performance liquid chromatography coupled to high-resolution tandem mass spectrometry (UHPLC-HRMS/MS) and Global Natural Products Social molecular networking (GNPS) were employed to map the complex metabolomic profile of these extracts. Specifically, we aim to quantify the concentrations of key neolignans in different plant organs, and to assess whether these quantitative variations correlate with antimycobacterial activity across leaves, stems, branches, and roots. Our objective is to guide the rational selection of plant parts as sources of pharmacologically active extracts or as starting materials for anti-tuberculosis lead compounds, rather than to propose immediate large-scale therapeutic use.

## 2. Results

### 2.1. Neolignan Quantification by HPLC-DAD-UV Analysis in Different Organs

**Figure 1:**
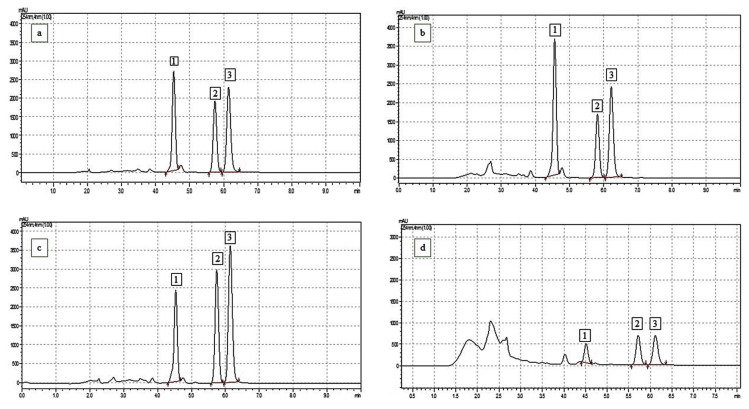
HPLC-DAD-UV chromatograms of crude extracts from different organs of *Piper rivinoides* Kunth. Peaks identification: (1) Conocarpan, (2) Eupomatenoid-5, (3) Eupomatenoid-6. Organs: (**a**) Leaves, (**b**) Branches, (**c**) Stems, (**d**) Roots. Red lines and arrows indicate the start and end of peak baseline.

The leaves showed the highest yield of crude hydroalcoholic extract (5.25%), followed by the roots (3.66%), stems (2.85%), and branches (2.74%). HPLC-DAD-UV analysis identified three major neolignans: (+)-conocarpan (CNC), eupomatenoid-5 (EUP-5), and eupoamtenoid-6 (EUP-6) in all extracts (chromatograms are presented in Figure 1, Table 1). The neolignan CNC was more concentrated in the leaves (4.73%), significantly higher than in the other organs (*p* < 0.01). EUP-6 exhibited lower and less variable concentrations across the organs, except in the roots, where no significant difference was observed compared to CNC. Overall, the roots displayed the lowest amounts of the analyzed compounds.

### 2.2. Organ-Specific Antimycobacterial Activity of Piper rivinoides Extracts

**Figure 2:**
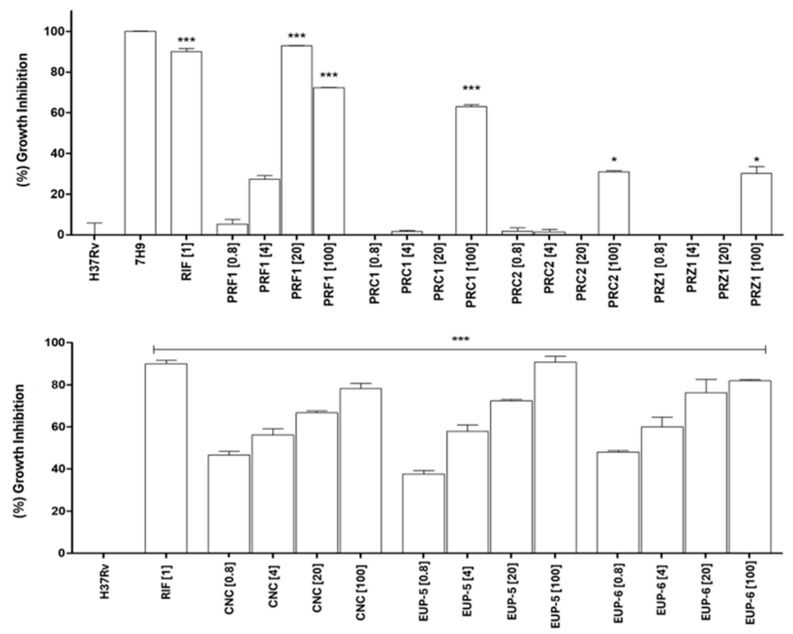
Inhibition of *Mycobacterium tuberculosis* H37Rv growth after treatment with crude extracts from different organs and isolated neolignanas of *Piper rivinoides*. MTT assay performed after 5 days of incubation with the samples. Positive control: *M. tuberculosis* H37Rv treated with rifampicin (RIF, 1 µg/mL); negative control: untreated *M. tuberculosis* H37Rv (7H9 medium only, 1 × 10^6^ CFU/mL). PRF1: crude leaf extract; PRC1: crude stem extract; PRC2: crude branch extract; PRZ1: crude root extract. CNC: conocarpan; EUP-5: eupomatenoid-5; EUP-6 eupomatenoid-6. The values in square brackets [ ] indicate the concentration: [0.8], [4], [20], and [100] μg/mL. Statistical analysis: One-way ANOVA followed by Tukey’s post hoc test. *** *p* < 0.001 and * *p* < 0.05 compared to the negative control. Results represent the mean ± standard error from triplicate experiments.

The inhibitory activity of hydroalcoholic extracts from *P. rivinoides* organs and isolated neolignans was evaluated against two *M. tuberculosis* strains: the virulent H37Rv and the hypervirulent M299. The percentage of growth inhibition is represented on the Y-axis of Figure 2, while the X-axis shows the different extracts—leaf (PRF1), branch (PRC1), stem (PRC2), and root (PRZ1)—along with positive and negative controls.

Among the extracts tested, the leaf extract (PRF1) showed the highest inhibitory activity against both strains. For the H37Rv strain, PRF1 showed a concentration-dependent inhibition, with 20 and 100 µg/mL producing significant effects, comparable to rifampicin. PRC1 also displayed strong inhibition, especially at 100 µg/mL, while PRR1 showed moderate activity. PRZ1 demonstrated the lowest activity, although 100 µg/mL resulted in improved inhibition.

Isolated neolignans CNC, EUP-5, and EUP-6 also showed significant antimycobacterial activity. Against the H37Rv strain, all compounds were active. CNC exhibited moderate inhibition, reaching approximately 40% at both 20 and 100 µg/mL. EUP-5 was more effective, inhibiting about 60% of bacterial growth at 20 µg/mL and up to 80% at 100 µg/mL, comparable to the positive control. EUP-6 also showed high efficacy, with approximately 80% inhibition at 100 µg/mL. These compounds exhibited activity comparable to, or even exceeding, rifampicin, with statistically significant results.

Minimum inhibitory concentration (MIC) values (Table 2) confirmed that PRF1 had the lowest MIC for both strains, indicating higher potency when comparable with the other samples.

**Figure 3:**
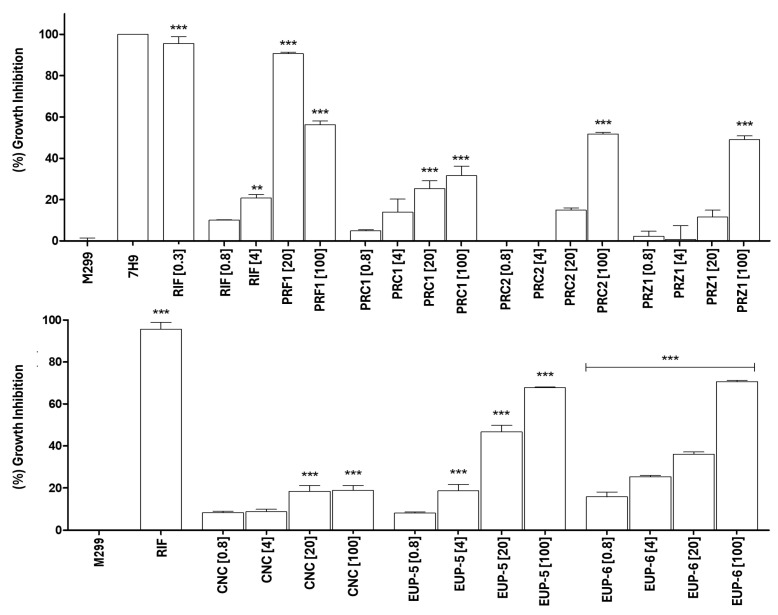
Inhibition of *Mycobacterium tuberculosis* M299 growth after treatment with crude extracts from different organs and isolated neolignanas of *Piper rivinoides*. MTT assay performed after 5 days of incubation with the samples. Positive control: *M. tuberculosis* M299 treated with rifampicin (RIF, 1 µg/mL); negative control: untreated *M. tuberculosis* M299 (7H9 medium only, 1 × 10^6^ CFU/mL). PRF1: crude leaf extract; PRC1: crude stem extract; PRC2: crude branch extract; PRZ1: crude root extract. CNC: conocarpan; EUP-5: eupomatenoid-5; EUP-6 eupomatenoid-6. The values in square brackets [ ] indicate the concentration: [0.8], [4], [20], and [100] μg/mL. For the positive control: RIF [1.0 µg/mL] (H37Rv) and RIF [0.3 µg/mL] (M299). Statistical analysis: One-way ANOVA followed by Tukey’s post hoc test. *** *p* < 0.001 and ** *p* < 0.01 compared to the negative control. Results represent the mean ± standard error from triplicate experiments.

Against the M299 strain, PRF1 at 20 µg/mL achieved the highest inhibition (~95%), followed by the PRZ1 at 100 µg/mL (~80%) and PRC1 at 20 µg/mL (~60%). Lower concentrations were generally less effective, except for PRF at 4 µg/mL, which showed moderate activity (~50%). Considering the pure neolignans EUP-5 and EUP-6 demonstrated strong inhibitory effects. EUP-5 inhibited ~60% of bacterial growth at 20 µg/mL and ~80% at 100 µg/mL, closely matching rifampicin. EUP-6 also achieved ~80% inhibition at 100 µg/mL. CNC showed moderate inhibition (~40%) at higher concentrations (Figure 3). All three neolignans exhibited low MIC_50_ values (Table 2), confirming their potent antimycobacterial activity against both *M. tuberculosis* strains.

**Figure 4:**
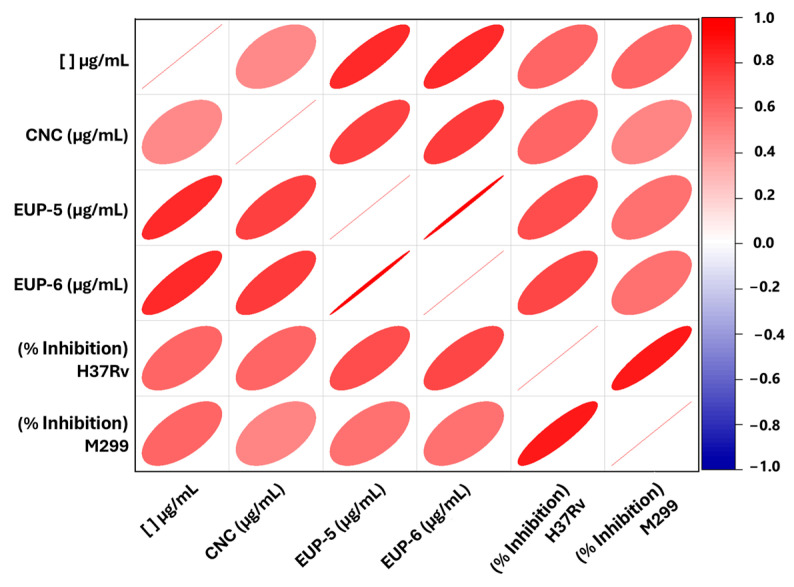
Correlation matrix showing the relationships between the concentrations ([ ] μg/mL) of neolignans (conocarpan = CNC; eupomatenoid-5 = EUP-5; eupomatenoid-6 = EUP-6) and the percentage of growth inhibition of *Mycobacterium tuberculosis* strains H37Rv and M299. Positive correlations are represented in red, with ellipse intensity and orientation indicating the strength and direction of the correlation. A stronger positive correlation is observed between EUP-5 and EUP-6 concentrations and the inhibition of the H37Rv strain. Correlation coefficients and significance levels are described in the main text.

Correlation analyses (Figure 4) indicated a moderate to strong relationship between the concentration of the three tested neolignans and the inhibition of *M. tuberculosis* H37Rv, with *p*-values < 0.05. This suggests a dose-dependent inhibitory effect, as higher compound concentrations were associated with greater bacterial growth inhibition. Among the compounds, EUP-5 (*r* = 0.716) and EUP-6 (*r* = 0.728) showed stronger correlations with the inhibition percentage than CNC (*r* = 0.607) for the H37Rv strain.

Although the correlation patterns were similar for both bacterial strains (H37Rv and M299), the relationships were only moderate for the M299 strain, with correlation coefficients of 0.494 for EUP-5; 0.564 for EUP-6; and 0.564 for CNC. These findings suggest that while all three neolignans showed inhibitory effects on both strains, their activity was more pronounced against the H37Rv strain.

### 2.3. Analysis of Crude Extracts by UHPLC-HRMS/MS

**Figure 5:**
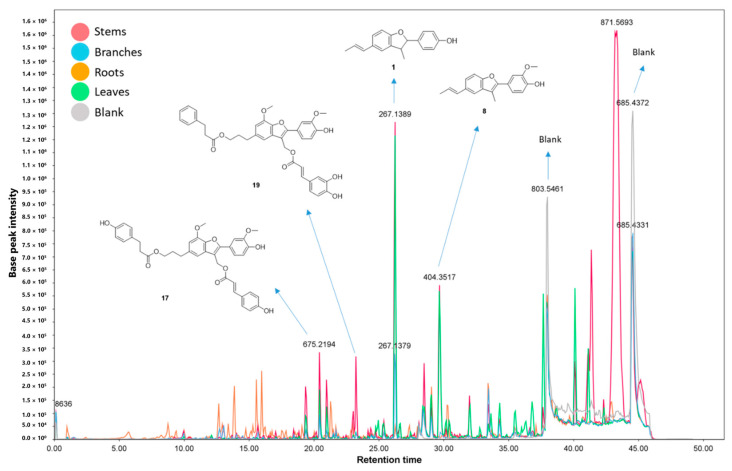
Base peak chromatogram obtained by Ultra-High Performance Liquid Chromatography coupled to High-Resolution Mass Spectrometry in tandem analysis, in positive ionization mode (ESI+) of stems, branches, leaves, and roots extracts of *Piper rivinoides*. Major annotated lignoids are highlighted in the chromatogram and their numbers agrees with the identification in the Table S1 and Figures S1–S10 (Supplementary Materials).

**Figure 6:**
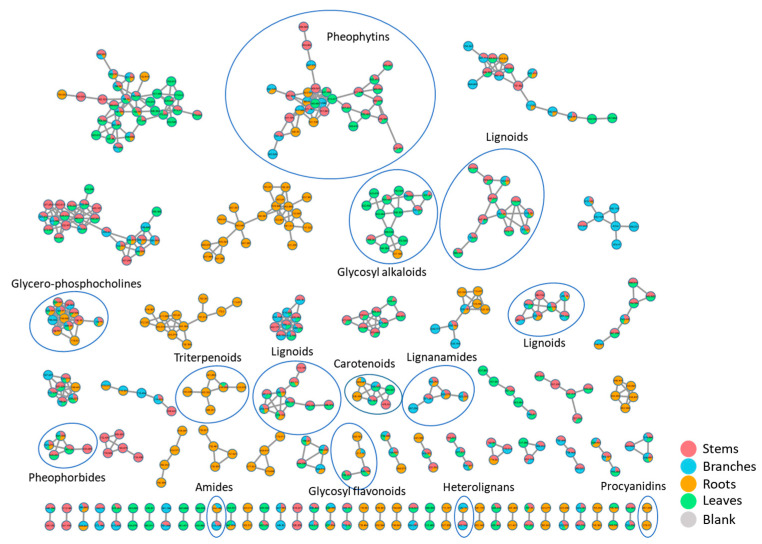
Molecular network for stems, branches, leaves, and roots extracts from *Piper rivinoides*, generated by Ultra-High Performance Liquid Chromatography coupled to High-Resolution Mass Spectrometry in tandem data, in positive ionization mode (ESI+). Only clusters containing at least two nodes are displayed. The Molecular families annotated in this study are highlighted by a circle.

**Figure 7:**
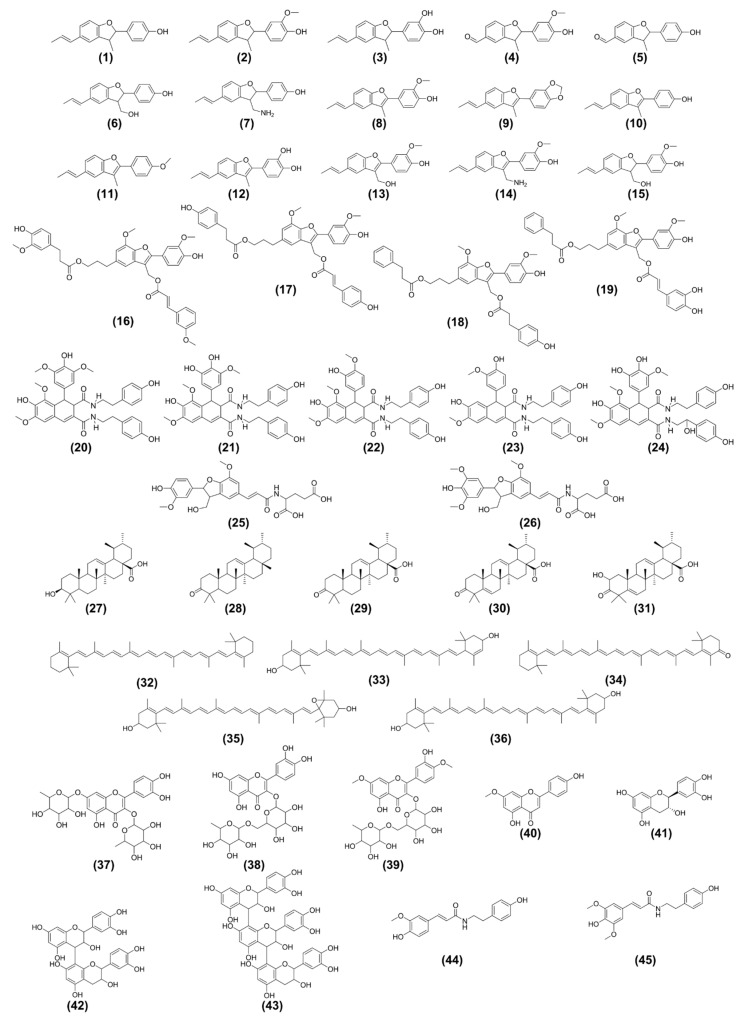
Main compounds annotated in the extracts from *Piper rivinoides*.

Extracts from different organs from *P. rivinoides* were analyzed using Ultra-High Performance Liquid Chromatography coupled to High-Resolution Mass Spectrometry in tandem (UHPLC-HRMS/MS), in positive electron spray ionization mode. The resulting data were processed through the GNPS2 (Global Natural Products Social Molecular Networking) platform for molecular network construction and compound annotation. Analysis of the chromatograms (Figure 5) and the molecular networks (Figure 6) revealed complex samples with a wide diversity of metabolites. The molecular networks comprised a total of **799** nodes, grouped into clusters also referred to molecular families. **Fourteen** molecular families were annotated, encompassing ions from different metabolite classes. In total, **58** compounds were putative identificated, including *neolignans*, *lignanamides*, *triterpenes*, *alkaloids*, *flavonoids*, *procyanidins*, *carotenoids*, *phosphocholines*, and *saccharides* (Figure 7). Additionally, clusters of pheophytins and pheophorbides (chlorophyll derivatives) were registered, although they will not be discussed in this work. It is worth noting that fourteen compounds were annotated for the first time.

## 3. Discussion

### 3.1. Chemical Organ-Specific Distribution

The chemical analysis of different organs of *P. rivinoides* revealed an organ-specific distribution of the neolignans EUP-5, EUP-6, and CNC. This distribution pattern aligns with the premise that distinct plant organs exhibit specific gene expression profiles, which directly influence the activation of metabolic pathways and enzyme production in particular tissues. Such variation results from adaptive processes and the complex ecophysiological interactions between the plant and its environment [24].

Although the occurrence of these neolignans has already been reported in *P. rivinoides* and other *Piper* species, such as *P. regnellii* (Miq.) C. DC. [25,26,27], and *P. solmsianum* C. DC. [28], this is the first investigation to demonstrate their specific distribution among different plant organs. This approach provides important insights into the spatial regulation of secondary metabolite biosynthesis with potential bioactivity.

Each plant organ responds uniquely to environmental stimuli, reflecting specific adaptations, such as distinct degrees of lignification, which are closely linked to the activation of the shikimate pathway—essential for the biosynthesis of lignoides [29]. Therefore, the variable presence of neolignans across plant organs may indicate metabolic specializations related to the physiological roles of each structure.

Although the specific ecological roles of neolignans remain poorly documented in the literature, their classification as phenolic compounds allows for the inference of important defensive functions. Phenolics are widely recognized for their antioxidant properties, including the neutralization of reactive oxygen species (ROS) and detoxification of hydrogen peroxide, thereby protecting plant tissues from oxidative damage. Moreover, these compounds are known to accumulate at infection sites to inhibit pathogen growth and restrict their spread at the point of entry [30].

The detection of all three neolignans (EUP-5, EUP-6, and CNC) in every analyzed organ, on the other hand, suggests a broad and multifunctional distribution of these compounds throughout the plant. This pattern may reflect the essential role of neolignans in plant defense against herbivory, pathogenic infection, and environmental stressors, functions that require their simultaneous presence in multiple tissues to ensure a coordinated and effective response [30,31].

In addition, the accumulation of lignoids in plants is often associated with responses to mechanical wounding or microbial invasion and is considered an adaptive phenotypic trait. These compounds reflect the plant’s ability to modulate its phenotypic plasticity in response to environmental stressors, thus contributing to its survival across diverse ecological settings [20,32,33,34]. In this context, it is plausible that lignoids accumulate in higher amounts in the organs most vulnerable to herbivory and other biotic and abiotic stresses, such as leaves and stems. Leaves are subject to a wide range of environmental challenges, including intense solar radiation, pathogen attacks, and herbivore pressure. As an adaptive response, these organs frequently develop specialized biochemical mechanisms, such as the accumulation of defensive secondary metabolites [35]. This selective allocation pattern is consistent with the optimal defense theory, which posits that plants strategically distribute their defensive resources to the most exposed and functionally critical tissues. The results of our study reflect and corroborate the accumulation of EUP-5, EUP-6, and CNC in the leaves. In fact, the concentrations of these benzofuran neolignans in the leaves were approximately twice those observed in the branches and stems, and nearly ten times higher than in the roots. This finding not only provides strong support for the optimal defense theory but also highlights the potential of these valuable compounds for biotechnological applications.

Therefore, the accumulation of neolignans across multiple plant organs, especially in the leaves, can be interpreted as part of a broad and integrated ecophysiological defense strategy, enabling the plant to cope effectively with a variety of environmental stresses [36,37].

### 3.2. Antimycobacterial Effects of Piper Rivinoides

Research on natural products represents a promising strategy for megadiverse countries such as Brazil, where underexplored plant species like *P. rivinoides* may serve as valuable sources of bioactive metabolites with therapeutic potential. Among major public health challenges, tuberculosis (particularly in its drug-resistant form) stands out due to its therapeutic complexity, which underscores the urgent need for new antimicrobial agents [38].

In this study, leaf extracts of *P. rivinoides* exhibited antimycobacterial activity comparable to rifampicin, which served as the positive control in the assay. Although extracts from other plant organs also demonstrated inhibitory effects, their efficacy was lower than that observed in the leaves, due to differential allocation of active compounds among plant tissues, which directly influences the biological effect. The variation in antimycobacterial activity of the extracts against *M. tuberculosis* strains H37Rv and M299 may be attributed to molecular differences that modulate bacterial susceptibility to neolignans. The H37Rv strain, a standard laboratory reference, showed greater sensitivity, likely due to its conserved cell wall structure and the absence of significant mutations in resistance mechanisms [39].

The chemical structure of neolignans, particularly the presence of phenolic and methoxylated functional groups, is directly related to their antimicrobial activity. Phenolic groups are known to generate reactive oxygen species (ROS), which induce oxidative stress and damage bacterial structures, while methoxylated groups enhance interactions with cell membranes, leading to cell wall destabilization and inhibition of mycobacterial growth [40,41,42,43].

On the other hand, the clinical strain M299, characterized by its hypervirulence and association with more severe clinical manifestations, exhibited lower sensitivity to the extracts. This resistance may be linked to the presence of mutations in critical genes, such as inhA, which is involved in the biosynthesis of mycolic acids, that is essential components of the mycobacterial cell wall, as well as the overexpression of efflux systems, such as pumps encoded by the *Rv1258c* gene, which facilitate the extrusion of bioactive substances from within the cell [44,45,46]. As mentioned before, neolignans and other phenolic compounds exhibit antimicrobial activity through multiple mechanisms, including interference with cell membrane permeability, thereby affecting ionic homeostasis and compromising bacterial viability. This effect is particularly relevant in resistant strains such as M299, whose cell wall displays structurally reinforced features. Evidence suggests that such compounds can penetrate these cellular barriers due to their affinity for membrane lipids [47]. Additionally, neolignans are notable for their ability to inhibit the enzyme InhA, which is involved in the biosynthesis of mycolic acids. Inhibition of this enzyme undermines the integrity of the cell wall, ultimately leading to cellular disintegration and bacterial death [47]. Other adaptive resistance mechanisms, such as reduced membrane permeability, modification of molecular targets, and alterations in the lipid composition of the cell wall, may also contribute to the decreased efficacy of the extracts against the M299 strain. Moreover, differential expressions of virulence genes may further reinforce these structural traits, thereby impeding the action of neolignans and other antimicrobial agents [46].

Plants of the genus *Piper* L. are widely recognized for their antimicrobial potential, exhibiting moderate to high activity against *M. tuberculosis* [15,26,48]. Numerous studies support the antifungal and antimycobacterial potential of extracts and fractions from *Piper* species. For example, *Piper abutiloides* Kunth showed antifungal efficacy, particularly in its hexane fraction, which exhibited minimum inhibitory concentrations (MICs) of 125 and 7.8 µg/mL against *Candida tropicalis* and *C. glabrata*, respectively, while other fractions did not show relevant activity [49]. Additionally, extracts from *P. diospyrifolium* Kunth and benzoic acid derivatives exhibited MICs of 125 µg/mL against the H37Rv strain [50], and root extracts from *P. corcovadensis* (Miq.) C.DC. demonstrated MICs ranging from 7.8 to 15.6 µg/mL against *M. tuberculosis* H37Rv, with no evidence of toxicity at the tested concentrations [51].

In the present study, leaf extracts from *P. rivinoides* exhibited even lower MICs against the H37Rv strain, suggesting superior efficacy compared to previously reported data. This potent antimicrobial activity may be associated with the high content of neolignans present in these extracts. Compounds such as EUP-5, EUP-3, and CNC, isolated from *P. regnellii* (Miq.) C.DC., have been extensively studied and are supported by robust experimental evidence regarding their therapeutic potential. Notably, the extract containing EUP-5 exhibited an MIC of 1.9 µg/mL against *M. tuberculosis* H37Rv [26]. EUP-5, isolated from *P. solmsianum* C.DC., also demonstrated antimycobacterial activity, with an MIC of 6.25 mg/L [28]. In addition to its action against mycobacteria, EUP-5 showed antifungal efficacy against various dermatophytes, such as *Microsporumcanis* and *Trichophyton mentagrophytes* [52]. Studies have also highlighted its synergistic effect with antitubercular drugs, such as rifampicin (RMP) and ethambutol (EMB), enhancing the therapeutic effects of these agents. Structure-activity relationship (SAR) analyses suggest that structural modifications of neolignans can significantly influence their efficacy, as well as their synergistic interaction with conventional drugs, thereby broadening their spectrum of action by targeting multiple bacterial metabolic pathways [53].

Other biological activities are also associated with the high content of the neolignan EUP-5 in plant extracts, including antitubercular [26,54], leishmanicidal [25], cytotoxic [55], activity against *Trypanosoma cruzi* [56,57,58], and antifungal action [59,60]. These reports reinforce the potential of incorporating EUP-5 into existing treatment regimens, enhancing therapeutic effects and allowing for dose reduction of conventional drugs, thereby minimizing adverse effects.

Regarding CNC, antibacterial activity has been reported in the literature against *Staphylococcus aureus* and *Escherichia coli* [61,62]. CNC is also notable for its leishmanicidal activity. Studies suggest that this neolignan can interfere with parasite viability, making it a promising therapeutic agent in the treatment of leishmaniasis [52]. An evaluation using a broad panel of pathogenic microbes involved in major community-acquired infectious diseases in Chile, including Gram-positive bacteria such as *Bacillus* sp., *Enterococcus* sp., *Listeria* sp., *Staphylococcus* sp., *Streptococcus* sp., and Gram-negative bacteria such as *Salmonella* sp., *Klebsiella* sp., *Enterobacter* sp., *Serratia* sp., *Pseudomonas* sp., *Yersinia* sp., *Vibrio* sp., *Citrobacter* sp., showed that CNC was among the main bioactive compounds [63]. Moreover, our research group [12] reported that CNC possesses biological activity against *Candida albicans*, *Leishmania amazonensis*, and *L. chagasi*, causative agents of candidiasis, cutaneous leishmaniasis, and visceral leishmaniasis, respectively. This was also confirmed by Dall’Igna et al., 2019 [64]. Although CNC exhibited good activity (MIC_50_ of 1.46 µg/mL), it showed a less effective inhibitory profile compared to EUP-6, suggesting that its activity may be related to differences in functional groups and electronic distribution, which can affect cellular permeability or the inhibition of enzymatic pathways [40,41].

EUP-6, in turn, has attracted attention for its antifungal properties and therapeutic potential [59,60]. Studies demonstrate its efficacy against several pathogenic fungal species, including *Microsporumcanis*, *M. gypseum*, *Trichophyton mentagrophytes*, *T. rubrum*, and *Epidermophyton floccosum*, all of which cause cutaneous infections. Its effectiveness against these strains underscores its potential as an antifungal agent. Although its exact mechanism of action is not yet fully elucidated, EUP-6 is believed to interfere with fungal membrane synthesis or inhibit enzymes essential to fungal growth [52]. The compound exhibited potent antifungal activity, with a MIC of 0.3 µg/spot against *Candida glabrata*.

In summary, this multidisciplinary study refines current knowledge of the chemistry of *P. rivinoides*, a species of medicinal and ritual relevance. UHPLC-HRMS/MS analyses of extracts from different organs suggested an abundance of neolignans, predominantly eupomatenoids, alongside additional constituents, some of which, to the best of our knowledge, have not previously been annotated for *P. rivinoides.* Compounds of biosynthetic interest were also tentatively annotated, including benzofuran-type neolignans bearing a nitrogen substituent at C-9, lignanamides, and triterpenes that appeared more prevalent in root extracts. In total, 58 features were putatively annotated; based on available literature and database searches, 14 appear to represent first reports. Collectively, these data illustrate the utility of LC–MS/MS and highlight the chemical diversity present in *P. rivinoides*. We also mapped the organ-level distribution of the major neolignans and observed in vitro antimycobacterial activity against *M. tuberculosis* H37Rv for the leaf extract and for the neolignans eupomatenoid-5, eupomatenoid-6, and conocarpan.

### 3.3. Chemical Investigation by UPLC-MS/MS

Compounds were putatively identified using GNPS molecular networking combined with manual inspection of MS/MS fragmentation patterns. Comparisons with public MS/MS databases were performed when possible, but these annotations remain tentative and are not fully confirmed.

Since the UPLC-MS/MS analyses are qualitative and were performed for the different organs, it makes sense to begin the discussion with the annotated compounds and then address the distribution pattern of the neolignans (+)-conocarpan, eupomatenoid-5 and eupomatenoid-6 that were quantified using standards previously isolated (see experimental).

Lignoids were annotated within the molecular networks across five distinct molecular families or clusters. In one cluster comprising seven nodes (Figure S1), the GNPS library annotated the neolignan conocarpan (**1**, *m*/*z* 267.1389 [M + H]+), one of the major ions observed in the chromatogram (Figure 5). The MS^2^ spectrum of conocarpan (**1**) predominantly displayed fragment ions at *m*/*z* 115 (C_9_H_7_^+^) and *m*/*z* 107 (C_7_H_7_O^+^), resulting from cleavages within the dihydrofuran ring. This neolignan has been previously reported in *P. rivinoides* and exhibits both antimicrobial and cytotoxic activity against oral carcinoma cells [12,16,65] (Figure S1). Also, this neolignan was used in this investigation to evaluate this distribution in different organs and its antimycobacterial activity. Manual propagation (i.e., expert-guided, network-based annotation propagation/manual inspection/manual annotation/propagation within a network family conditioned on structural/chemo-typic coherence, consistency of diagnostic ions, and adduct/isotope/neutral-loss relationships), analysis of the remaining ions in this molecular family led to the annotation of six additional minor compounds (**2**–**7**) (Figure S2), all with mass errors below 5 ppm. Among them, a non described neolignan named **rivinoidine A** (**7**) was annotated, and no isolation or NMR confirmation has been performed, with a molecular formula of C_18_H_20_NO_2_ and *m*/*z* 282.1507 [M + H]+. Rivinoidine A (**7**) features an NH_2_ group in its structure, and the presence of the same fragment ions (*m*/*z* 115 and *m*/*z* 107) in its MS^2^ spectrum suggests that the amino group is attached to the C9 carbon (Figure 7 and Figure S11). The biosynthesis hypothesis of this compound will be discussed bellow. Within this molecular family, two compounds bear a carbonyl group at the C7′ (compounds **4** and **5**, Figure S1), which likely originates from a biosynthetic β-oxidation of the C3 chain (propenyl group). This transformation results in a characteristic fragment ion at *m*/*z* 103 (C_7_H_3_O^+^).

Another molecular family of lignoids (Figure S2) was identified through manual annotation as eupomatenoid 5 (**8**) (*m*/*z* 295.1331 [M + H]+). The MS^2^ spectrum displayed fragment ions consistent with reference data from the MassBank of North America [66] for compound **8**. The main MS^2^ product ion at *m*/*z* 128.06 (C_10_H_8_^+^) suggests a radical-driven cleavage within the furan ring of eupomatenoid 5 (**8**). In addition, three known lignoids, eupomatenoids-3 (**9**, *m*/*z* 293.1191 [M + H]+), eupomatenoid-6 (**10**, *m*/*z* 265.1236 [M + H]+), and eupomatenoid-15 (**11**, *m*/*z* 279.1391 [M + H]+) were also annotated, along with three newly suggested eupomatenoid derivatives. All annotations were supported by exact mass errors below 5 ppm. These putative identificated new compounds include: (a) an hydroxylated derivative of eupomatenoid-6 at C5 of the aromatic ring (**12**, *m*/*z* 281.1186 [M + H]+), that may result of an hydroxylation after eupomatenoid-6 formation (by an oxidase enzyme); (b) an hydroxylated derivative of eupomatenoid-5 at C9 (**13**, m/z 311.1281 [M + H]+) that may be formed by a CYP450 oxidation of the methyl group; (c) an eupomatenoid-5 analog bearing an NH_2_ group at position C9, named as **rivinoidine B** (**14**, *m*/*z* 310.1448 [M + H]+), that is an analougue of rivinoidine A (**7**) (Figure S3). The substitution positions were proposed based on plausible mass fragmentation patterns of these tentative identificated compounds.

**Figure 8:**
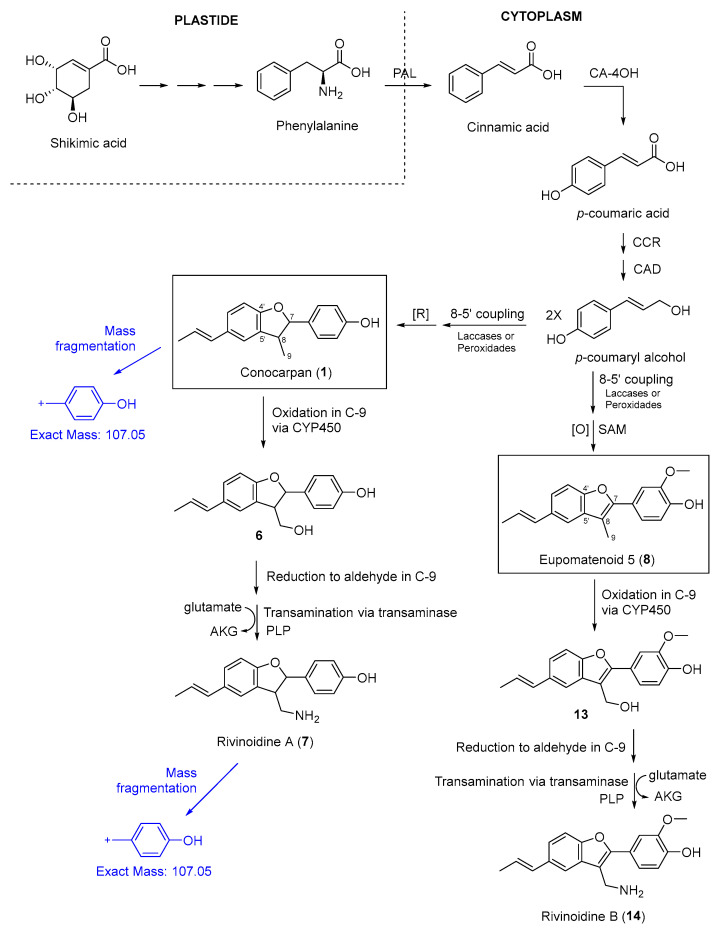
Biosynthetic proposal for formation of rivinoidines A and B, as well as main fragmentation ions (in blue). PAL = phenylalanine ammonia-lyase; CA-4OH = cinnamic acid 4-hydroxylase; CCR = *p*-coumaryl-CoA reductase; CAD = cinnamyl alcohol dehydrogenase; SAM = S-adenosyl-methionine; [O] = oxidation; [R] = reduction; AKG = α-ketoglutarate; PLP = pyridoxal phosphate.

The compounds rivinoidine A (7) and rivinoidine B (14) are tentatively annotated as amino-benzofuranone neolignans and are structurally distinctive; their putative biosynthetic pathway is shown in Figure 8, as well as the annotated family for these compounds in Figures S1 and S2. As extensively discussed in the literature [67], phenylalanine biosynthesis originates from shikimic acid within the plastids. The deamination of phenylalanine by phenylalanine ammonia-lyase (PAL) yields cinnamic acid in the cytoplasm. Cinnamic acid (in its conjugated with de enzyme Coenzyme A, cinnamoyl-CoA) is subsequently hydroxylated at C-4 position by cinnamic acid 4-hydroxylase (CA-4OH), producing *p*-coumaric acid (or *p*-coumaryl-CoA). This compound is then converted sequentially by the enzymes *p*-coumaryl-CoA reductase (CCR) and cinnamyl alcohol dehydrogenase (CAD) into *p*-coumaryl alcohol. Two molecules of *p*-coumaryl alcohol undergo oxidative coupling at the 8–5′ positions, mediated by laccases or peroxidases, followed by reduction, yielding conocarpan (**1**). Conocarpan is then oxidized at the C-9 position by cytochrome P450 enzymes (CYP450s) or other oxidases, leading to the formation of a hydroxylated derivative at this site, which corresponds to a compound also identified in the extracts (neolignan **6**). This C-9 hydroxylated neolignan is further oxidized to an aldehyde and subsequently undergoes a transamination reaction catalyzed by aminotransferases. In this step, an amino group is transferred from a donor amino acid (likely glutamate, which is converted to α-ketoglutarate) resulting in the introduction of an NH_2_ group at the C-9 position and the formation of rivinoidine A. Analogous transformations occur with eupomatenoid-5 (**8**), which follows the same oxidative sequence at C-9 and subsequent transamination, ultimately yielding rivinoidine B. Since the structural annotations were performed by HRMS/MS, it is not possible to determine the absolute configuration of rivinoidine A (positions 7 and 8), as well as some other tentatively proposed neolignan compounds (**2**, **3**, **4**, **5**, **6** and **15**) here annotated (Figure 7).

Figure 7 also highlights one of the main fragment ions in the mass spectra of conocarpan (*m*/*z* 151, C_9_H_7_^+^) and rivinoidine A (*m*/*z* 151, C_9_H_7_^+^). The proposed fragmentation pathways for rivinoidine A (**7**) and B (**14**) are detailed in the Supplementary Material, Figures S11 and S12, respectively. For rivinoidine A (**7**), in addition to the *m*/*z* 107 fragment (Figure 8), the most relevant fragments for establishing the proposed structure were annotated. The *m*/*z* 273, *m*/*z* 133, and *m*/*z* 107 fragments support the presence of a hydroxyl group on the aromatic ring, in a position analogous to that of conocarpan (**1**). The *m*/*z* 224 and *m*/*z* 195 fragments indicate the presence of an amino group on the methyl substituent at position 9 (Figure S11). For rivinoidine B (**14**), a benzofuran-type neolignan, the most relevant fragments for structural elucidation were *m*/*z* 239, *m*/*z* 210, and *m*/*z* 165, which support the presence of hydroxyl and methoxyl groups on the aromatic ring in a position analogous to that of eupomatenoid 5 (**8**). The *m*/*z* 263 and *m*/*z* 131 fragments indicate the presence of an amino group on the methyl substituent at position 9.

The other compounds annotated as new are analogs of known natural compounds described in the literature, therefore their fragmentation pathways were not detailed in this article.

These eupomatenoids from this two families (Figures S1 and S2) were variably distributed across the studied plant organs, with the exception of rivinoidine B (**14**), which was detected exclusively in the stem. Eupomatenoids-3 (**9**), 5 (**8**), and 6 (**10**) have previously been reported in *P. rivinoides* [12], as well as eupomatenoid-5 (**8**) and eupomatenoid-6 (**10**) were used in this study to proceed with the distribution analysis in different organs and their antimicrobial activity.

A third molecular family, composed of lignans (**15**–**19**), was identified within the molecular network and enabled the annotation of five compounds primarily present in the leaves, branches, and stems of *P. rivinoides*. The two novel-suggestions benzofuran neolignans 9-hydroxy-parakmerin A (**15**, *m*/*z* 647.2620 [2M + Na]+) and 9-hydroxy-eupomatenoid-5 (**13**, *m*/*z* 643.2300 [2M + Na]+) were annotated in their dimeric ion forms, with MS^2^ spectra showing their corresponding monomeric ions as major product ions. Compound **13** had also been previously annotated in another molecular family as its protonated adduct [M + H]+. Within the same molecular family, a subgroup of [M + Na]+ ions allowed the manual annotation of four additional previously unreported neolignans. The tentatively identified compounds (**16**–**19**), named here as eupomatenoids-A-D, are structural analogs of the known lignans boehmenan and boehmenan X [68,69,70]. The main MS^2^ fragment ion characteristic of this molecular family, *m*/*z* 349.10 (C_19_H_18_NaO_5_+), results from the loss of *p*-cumaric and cinnamic acid-derived moieties. Indeed, eupomatenoids-A-D are strutucture related to previously annoted benzofuran neolignans eupomatenoids-5 (**8**), 6 (**10**), and 3 (**9**), but with the difference of *p*-cumaric and/or cinnamic acid-derived moieties at C9 and C9′. These acids moieties C_6_–C_3_ likely possibly are incorporated in the benzofuran neolignan pathway through esterification of the carboxyl moiety (H-O-C=O) with the hydroxyl group (HO-) at C-9 and C-9′.

A known lignanamide, flavifloramide B (**20**, *m*/*z* 685.2738 [M + H]+), was annotated by the GNPS library within a molecular family comprising five nodes. The MS^2^ spectrum of the adduct ion showed 12 shared peaks with the reference spectrum from the database. The major fragment ions correspond to the loss of two tyramine moieties and subsequent elimination of CO (*m*/*z* 383), followed by the loss of a methoxy group via CH_3_OH elimination (*m*/*z* 351). Within the same molecular family, four additional lignanamides (**21**–**24**) were manually annotated, all displaying a similar fragmentation pattern. Among them, three are known compounds, and one is a newly putatively identified derivative, 7″-hydroxy-flavifloramide A (**24**, *m*/*z* 687.2541 [M + H]+). Compound **24** exhibited the same major fragment ions as flavifloramide A (**21**, *m*/*z* 671.2582 [M + H]+), with a mass difference of 16 u, suggesting the presence of an additional hydroxyl group at C7″ on one of the tyramine units. Flavifloramides A (**21**) and B (**20**) have previously been reported in *Piper flaviflorum* [71] (Figure S4). The lignanamides annotated in this study were detected in the roots, branches, and stems of *P. rivinoides*.

The heterolignan **25** (*m*/*z* 502.1680 [M + H]+), annotated by the GNPS library within a two-node cluster, was detected in the branches and stem of *P. rivinoides*. The other compound in this molecular family, **26** (*m*/*z* 532.1800 [M + H]+), was tentatively identified through manual propagation and was registered in the plant’s branches and roots (Figure S5). Both compounds are newly-suggested reported in the *Piper* genus and displayed a base ion at *m*/*z* [M + H − 137]+ in their MS^2^ spectra. This fragment was attributed to amide bond cleavage followed by the elimination of methanol and water neutral fragments. Compound **26** has been tentatively described for the first time.

Ursolic-type triterpenes were primarily annotated in the roots of *P. rivinoides*, based on a molecular cluster comprising six nodes. Ursolic acid (**27**, *m*/*z* 439.3566 [M + H]+) was identified by the GNPS library, with eight shared peaks compared to the reference MS^2^ spectrum, including the major fragment ions at *m*/*z* 203.18 (C_15_H_23_^+^) and *m*/*z* 189.16 (C_14_H_21_^+^). Ursolic acid (**27**) has previously been reported in species of the *Piper* genus [72,73]. Within the same molecular family, two additional known triterpenes were manually annotated: α-amyrenone (**28**, *m*/*z* 425.3793 [M + H]+) and ursonic acid (**29**, *m*/*z* 455.3505 [M + H]+). Moreover, two ursane-5,6-dehydro derived triterpenes were annotated, compound **30** (*m*/*z* 469.3321 [M + H]+) and compound **31** (*m*/*z* 453.3373 [M + H]+). Compound **31** is further suggested to contain a hydroxyl group at the C2 position, based on the proposed biosynthetic pathway of this class. To the best of our knowledge, compounds **30** and **31** are novel pentacyclic triterpenes, both structurally related to ursonic acid (**29**). It is interesting to note that four out of the five identified triterpenes were detected in the roots (**28**–**31**) (Figure S6). Only compound ursolic acid (**27**) was annoted in the leaves and branches, besides in the roots.

A carotenoids molecular Family, comprising six nodes, was also formed based on the molecular networking analysis. The GNPS library annotated β-carotene (**32**) as the molecular ion (*m*/*z* 536.4347, [M]+), showing seven shared peaks compared to the reference MS^2^ spectrum. Within the same node cluster, lutein (**33**, *m*/*z* 568.4259 [M]+) was manually annotated as the molecular ion (M+), while echinenone (**34**, *m*/*z* 551.4217 [M + H]+), antheraxanthin (**35**, *m*/*z* 585.4278 [M + H]+), and zeaxanthin (**36**, *m*/*z* 569.4357 [M + H]+) were annotated as protonated adduct ions [M + H]+. Published data indicate that electrospray ionization (ESI) commonly produces molecular ions (M+) for β-carotene and lutein [74] (Figure S7).

Three glycosylated flavonoids were also annotated in the extracts of *P. rivinoides*, grouped into a small molecular family of four nodes. Quercetin 3,7-dirhamnoside (**37**, *m*/*z* 595.1650 [M + H]+) and rutin (**38**, *m*/*z* 611.1600 [M + H]+) were annotated by the GNPS library with mass errors below 5 ppm and MS^2^ fragment ions matching reference database spectra. Ombuin-3-*O*-rutinoside (**39**, *m*/*z* 639.1990 [M + H]+) was annotated through manual propagation. The MS^2^ spectra of these glycosylated flavonoids showed fragment ions corresponding to the aglycone flavonoid, confirming the loss of sugar moieties [75]. Additionally, the flavonoids genkwanin (**40**, *m*/*z* 285.0760 [M + H]+), catechin (**41**, *m*/*z* 291.0864 [M + H]+), and procyanidins B2 (**42**, *m*/*z* 579.1496 [M + H]+) and C1 (**43**, *m*/*z* 867.2129 [M + H]+) were annotated by the GNPS library with MS^2^ fragmentation consistent with database entries (Figure S8). Flavonoids and their glycosylated derivatives are commonly reported in species of the *Piper* genus [76,77,78].

Moupinamide (**44**, *m*/*z* 314.1390 [M + H]+) and *N*-*trans*-sinapoyltyramine (**45**, *m*/*z* 344.1490 [M + H]+) were tentatively identified within a two-node cluster. Moupinamide (**44**), previously reported in the *Piper* genus [79,80], was detected in the branches and stem of *P. rivinoides*, while *N*-*trans*-sinapoyltyramine (**45**) was found in the branches and roots of the plant (Figure S9). The MS^2^ fragmentation spectra of these amides exhibited a characteristic fragment at *m*/*z* 121.06, corresponding to the 4-ethylphenol moiety formed by amide bond cleavage followed by ammonia (NH_3_) elimination [81].

A molecular family comprising 11 glycero-phosphocholines (**46**–**56**) was annotated by the GNPS library (Figure S10). These compounds were distributed variably across all the organs studied in *P. rivinoides*. Glycero-phosphocholines are commonly found in plant cells, particularly within their membranes [82]. The MS^2^ fragmentation spectra exhibited a characteristic ion at *m*/*z* 184.07, corresponding to the phosphocholine moiety, which is typical for this compound class [83].

In addition to the molecular families described, two saccharides were also annotated by the GNPS library. Stachyose (**57**, *m*/*z* 689.2081 [M + Na]+) and raffinose (**58**, *m*/*z* 527.1560 [M + Na]+) were detected in the roots and leaves of *P. rivinoides*. These saccharides are commonly found in plants, typically in seeds, where they play crucial roles in regulating plant responses to various abiotic stresses [84]. The MS^2^ fragmentation spectra of both compounds showed the adduct ion [M + Na − 162]+ as the base peak, indicating the loss of a glycosyl group.

Although further studies, including toxicity evaluation and pharmacokinetic analyses, are required before any therapeutic application, the results suggest that *P. rivinoides* leaves, which exhibited the highest antimycobacterial activity, may represent a promising source of bioactive compounds for future drug development.

## 4. Materials and Methods

### 4.1. Plant Materials

*Piper rivinoides* Kunth (roots, stems, branches, and leaves) were collected at 9:00 a.m. in January 2022, during the summer season, under collection authorization no. 07/0002.007362/2021. The sampling was carried out in Pedra Branca State Park, located in the West Zone of the city of Rio de Janeiro, Brazil (22°58′12″ S, 43°14′30″ W, elevation 452 m). The park is recognized as the largest urban forest in the world, with a humid tropical climate and no defined dry season. A pool of five specimens with the same morphological characteristics was selected to increase the genetic variability of the samples. Botanical identification was performed by Dr. George Queiroz de Azevedo from the Rio de Janeiro Botanical Garden Research Institute. A voucher specimen was deposited at Herbarium of the Rio de Janeiro Botanical Garden (RB) under the number RB 861754. This study was registered in the National System for Management of Genetic Heritage and Associated Traditional Knowledge (SisGen) under registration number AE4E953.

### 4.2. Extract Preparation

Plant organs were stored at −20 °C and subsequently dry-frozen to ensure dehydration without heat, preserving thermosensitive compounds. The dried plant material was cut with scissors, sieved, and 20.0 g of the resulting material was extracted in 200 mL of a hydroalcoholic solution (ethanol: water, 9:1 *v*/*v*), according to [27]. Hydroalcoholic extraction (EtOH:H_2_O 7:3 *v*/*v*) was selected as it efficiently extracts both polar and moderately non-polar metabolites, providing a representative metabolomic profile of the plant material. This solvent system is also consistent with traditional preparations used for medicinal purposes. Briefly, the mixture was subjected to ultrasound-assisted extraction in an ultrasonic bath (Unique USC1400, 40 KHz, 120 W, Sanders, Santa Rita, Brazil) at 22 °C for 30 min [85]. After extraction, the solution was filtered through qualitative cellulose filter paper (Sigma-Aldrich^®^, São Paulo, Brazil) and air-dried under a fume hood. The resulting semi-dried extract was dry-frozen again to remove residual water, until constant weight. Extraction yield was calculated based on the dry weight of the extract relative to the initial weight of plant material (*w*/*w*, % yield).

### 4.3. HPLC-DAD-UV Analysis

Considering the initial extract concentration (10 mg/mL), the percentage values of the compounds were calculated relative to the dry weight of the crude extract used for analysis. Quantification of the neolignans eupomatenoid-5 (EUP-5), eupomatenoid-6 (EUP-6), and (+)-conocarpan (CNC) was performed by HPLC-DAD-UV (Nexera Prominence HPLC system, Shimadzu^®^, Kyoto, Japan) using analytical calibration curves constructed for each pure isolated compound (chromatographic purity > 95%, determined by HPLC-DAD-UV), kindly provided by Dr. André Mesquita Marques, previously isolated from *P. rivinoides*. The procedures for extraction, purification, isolation, and structural elucidation of these neolignans are detailed in [12]. Stock solutions (400 μg/mL) of each compound were prepared in methanol and stored under refrigeration (5 °C), protected from light. The neolignans (+)-conocarpan, eupomatenoid-5, and eupomatenoid-6 were quantified by HPLC–DAD–UV using external calibration (five points; 12.5–200 μg mL^−1^) with purified isolated standards, following the previously developed and validated method by Felisberto et al. [27], which assessed selectivity, linearity, precision, accuracy/recovery, and LOD/LOQ (r^2^ > 0.99). Quantification was based on peak area, with correction by the dilution factor when applicable, such as for the leaf samples, and results were expressed as % (*w*/*w*) relative to the crude extract. Linear regression equations for the quantification of each neolignan were calculated by the least-squares method and are as follows: (a) eupomatenoid-5: concentration = (ABS − 25,605)/96,143; (b) eupomatenoid-6: concentration = (ABS − 17,370)/129,549; (c) (+)-conocarpan: concentration = (ABS − 478,517)/76,688. In these equations, concentration is given in μg·mL^−1^ and ABS is the absorbance obtained by HPLC–DAD–UV analysis. Considering the initial amount of extract used, percentage values of the compounds were then calculated. Response curves used for quantification were established based on the average and standard deviation of the slope and y-intercept from three replicates with *r*^2^ values greater than 0.99 [27]. For neolignan content analysis, 10 mg of each crude extract was solubilized in 1 mL of HPLC-grade methanol (Tedia, Brazil), followed by sonication for 15 min to ensure complete dissolution. The solution was then filtered through a 0.45 μm PVDF filter (Sigma-Aldrich^®^, São Paulo, Brazil) and analyzed by HPLC-DAD-UV. The analyses were conducted using a Nexera Prominence HPLC system (Shimadzu^®^, Kyoto, Japan) equipped with a binary pump LC-20AT, autosampler SIL-20AC, DAD-UV-VIS detector SPD-M20A, degasser DGU-20As, and column oven CTO-20AC. The chromatographic column used was a Supelco Ascentis C18 (250 mm × 4.6 mm i.d., 5 μm particle size). HPLC condition analysis was the same as published before [27]. Data acquisition and processing were performed using Shimadzu LabSolutions^®^ V2.04 software.

### 4.4. UHPLC-HRMS/MS and Molecular Networking Analysis

Extract solutions were prepared at a final concentration of 5 mg/mL in spectroscopic-grade methanol (Tedia, Brazil) and analyzed by LC-MS/MS at the Analytical Platform of Farmanguinhos/ FIOCRUZ, with support from Dr. André Mesquita Marques. Analyses were performed using a Nexera UHPLC system (Shimadzu^®^, Kyoto, Japan) coupled to a High-Resolution Mass Spectrometer (UHPLC-HRMS/MS, Impact, Bruker, Billerica, MA, USA) equipped with Electrospray Ionization (ESI) and detection by a Q-TOF Mass Spectrometer. Spectra were acquired in both positive and negative ionization modes, using full scan mode from 40 to 2000 *m*/*z*, with fragmentation of the five most intense ions per retention time (tR). A 1 μL aliquot of each sample (5000 ppm) was injected into an Acquity BEH C18 column (100 mm × 2.1 mm i.d., 1.7 μm particle size, Waters, Milford, MA, USA) at 40 °C, with a mobile phase flow rate of 0.3 mL/min. The mobile phases consisted of A: ultrapure water with 0.1% formic acid (Milli-Q system) and B: acetonitrile with 0.1% formic acid (HPLC grade, Sigma-Aldrich^®^, São Paulo, Brazil). The elution gradient started with 5% B for the first 5 min, followed by a linear gradient to 100% B over 39 min, which was held for an additional 5 min. The mass spectrometer parameters were as follows: capillary voltage, 4500 V; nebulizer gas (N_2_) pressure at 4.0 bar; desolvation gas flow of 10 L/min; transfer capillary temperature of 200 °C; and end plate offset at −500 V [86]. Data acquisition was performed using DataAnalysis^®^ software 4.2 version (Bruker Daltonics, Billerica, MA, USA), and raw files were converted to mzXML format using MSConvert software 1.0 version (Palo Alto, CA, USA).

For molecular networking generating, data from MS/MS spectra were processed using the GNPS online workflow (https://ccms-ucsd.github.io/GNPSDocumentation/, accessed on 10 July 2025). Fragment ions within ±17 Da of the precursor *m*/*z* were removed. Spectra were window-filtered, retaining only the top 6 fragment ions within ±50 Da windows across the spectrum. The precursor and fragment ion mass tolerances were both set to 0.02 Da. Molecular networks were generated and visualized in Cytoscape^®^ 3.18 version using an organic layout. Edges in the network were filtered to retain those with a cosine score above 0.65 and at least 4 matched peaks. Edges were only maintained if each node appeared in the other’s top 10 most similar nodes. The maximum molecular family size was limited to 100 nodes, and edges with the lowest scores were removed until the family met this criterion. All matches between network spectra and library spectra had cosine scores above 0.7 and a minimum of 4 shared peaks, following the procedures described by [87,88]. The complete molecular networking workflow and results can be accessed at: https://gnps.ucsd.edu/ProteoSAFe/status.jsp?task=acc800910da64f60ac9910f689fdaceb (accessed on 10 July 2025). Compounds suggested by GNPS were evaluated for inclusion based on: natural origin; previous reports in *Piper* species or other angiosperms; or, in the case of putatively novel compounds, structural similarity to known natural products and biosynthetic approach. It is important to note that GNPS-based dereplication only provides putative identifications, particularly due to the presence of chiral centers in most natural products, which precludes definitive structural elucidation solely by MS data.

### 4.5. Biological Assay

#### 4.5.1. Mycobacterium Tuberculosis Growth

The *M. tuberculosis* strains used for evaluation included the standard virulent strain H37Rv (ATCC 25618) and the hypervirulent Beijing M299 strain, previously isolated from tuberculosis patients in Mozambique and kindly provided by Dr. Philip Suffys (Oswaldo Cruz Foundation, Rio de Janeiro, Brazil). Both strains were cultured in 7H9 medium (BACTO), supplemented with 10% albumin, dextrose, and catalase (Sigma-Aldrich^®^, São Paulo, Brazil), and 0.05% Tween 80. Cultures were incubated at 37 °C in a 5% CO_2_ atmosphere using a water-jacketed incubator (Thermo Fisher Scientific, Waltham, MA, USA) at 37 °C, under biosecurity level 3 containment conditions until the exponential growth phase.

#### 4.5.2. Mycobacterium Tuberculosis Activity

The antimycobacterial activity of the samples was evaluated using a 96-well microplate tetrazolium salt assay at final concentrations of 0.8, 4, 20, and 100 μg/mL. For the assay, suspensions of *M. tuberculosis* H37Rv and Beijing M299 strains were prepared by mixing 300 μL of bacterial culture with 7.2 mL of 7H9 broth supplemented with 10% ADC, yielding approximately 3 × 10^7^ CFU/mL. Cultures were incubated at 37 °C with 5% CO_2_ until reaching the logarithmic growth phase. Bacterial concentration was standardized by turbidity, monitored by spectrophotometry at 600 nm (Hitachi U-1100). At the log phase, 50 μL of the bacterial suspension (1 × 10^6^ CFU/well) was dispensed into each well of a 96-well plate. Test samples (extracts or isolated compounds) were previously diluted in 7H9 + ADC at twice the desired final concentration, and 50 μL was added to each well containing the bacterial suspension. Plates were sealed and incubated at 37 °C and 5% CO_2_ for 5 days.

After incubation, 10 μL of MTT solution (5 mg/mL in sterile phosphate-buffered saline) was added to each well. After 3 h, 100 μL of lysis buffer (20% SDS *w*/*v* and 50% DMF in distilled water, pH 4.7) was added. The absorbance was read at 570 nm using a microplate spectrophotometer. Rifampicin was used as a positive control at 1 μg/mL for the H37Rv strain and 0.3 μg/mL for the M299 strain. The negative control consisted of wells containing only the bacterial suspension without treatment. The percentage of growth inhibition was calculated using the formula:

100 − (O.D. sample − O.D.C^+^) × 100/(O.D.C^−^ − O.D.C^+^) (1)

where O.D. sample is the absorbance of the test sample (extract or pure compound), O.D.C^+^ is the absorbance of the positive control (rifampicin-treated), O.D.C^−^ is the absorbance of the negative control (untreated bacteria).

### 4.6. Statistical Approaches

The chromatographic data were subjected to parametric statistical analysis using ANOVA (*p* < 0.05) with the software Statistica, version 13 (StartSoft Inc., Tulsa, AZ, USA), and Origin Pro, version 2024 (Origin Lab, Northampton, MA, USA), to compare the means obtained among the extracts. The means were compared using Tukey’s test at a 5% significance level [89].

For the antimycobacterial assays, descriptive statistics were employed, with the mean and standard deviation calculated for each group. A one-way ANOVA was then performed to compare the group means, followed by Tukey’s post hoc test for multiple comparisons, adopting a 5% significance level (*p* < 0.05) [89].

## Figures and Tables

**Table 1:** Crude Extract Yield and Neolignan Concentrations in Different Organs of *Piper rivinoides* Kunth.

Plant Organ	Extract Yield (% *w*/*w*)	CNC (%)	EUP-5 (%)	EUP-6 (%)
Leaves	5.25	4.73 ± 1.98 ^a^	3.81 ± 0.44 ^b^	2.12 ± 0.20 ^f^
Branches	2.74	2.93 ± 2.90 ^e^	2.01 ± 0.40 ^c^	0.94 ± 0.32 ^h^
Stems	2.85	2.40 ± 2.40 ^d^	3.10 ± 0.51 ^g^	1.70 ± 0.45 ^i^
Roots	3.66	0.40 ± 0.20 ^j^	0.60 ± 0.66 ^k^	0.41 ± 0.10 ^k^

Legend: Values are mean ± SD (*n* = 3 independent extracts). Extract yield is expressed as % (*w*/*w*) of crude extract relative to dried plant material. CNC, EUP-5, and EUP-6 are expressed as % (*w*/*w*) of analyte in the crude extract determined by external calibration. Within each column, means followed by different superscript letters (a–k) differ significantly among organs (one-way ANOVA followed by Tukey’s HSD; α = 0.05). Means sharing the same letter do not differ significantly. Abbreviations: CNC = (+)-conocarpan; EUP-5 = eupomatenoid-5; EUP-6 = eupomatenoid-6.

**Table 2:** Organ-Specific Minimum Inhibition Concentration Antimycobacterial Activity of *Piper rivinoides* Extracts and Isolated Neolignans.

Sample	H_37_Rv MIC_50_ (µg/mL)	H_37_Rv MIC_90_ (µg/mL)	M299 MIC_50_ (µg/mL)	M299 MIC_90_ (µg/mL)
PRF1	7.16	65.61	8.99	82.41
PRC1	99.22	NC	>100	NC
PRC2	>100	NC	101.7	NC
PRZ1	>100	NC	112.9	NC
CNC	1.46 ± 1.23	NC	>100	NC
EUP-5	2.23 ± 1.13	~100	28.79 ± 1.14	NC
EUP-6	1.04 ± 1.23	NC	31.96 ± 1.19	NC

Legend: CNC = (+)-Conocarpan; EUP-5 = Eupomatenoide-5; EUP-6 = Eupomatenoide-6; PRF1 = Leaves, PRC1 = Branches, PRC2 = Stems, PRZ1 = Roots. NC = Non calculated.

## Data Availability

The complete molecular network and other parameters used for its construction are publicly available at the GNPS job link: https://gnps.ucsd.edu/ProteoSAFe/status.jsp?task=acc800910da64f60ac9910f689fdaceb, accessed on 10 July 2025.

## Supplementary Materials

The following supporting information can be downloaded at: https://www.mdpi.com/article/10.3390/molecules30244682/s1, Supplementary materials include Table S1: UHPLC-HRMS-MS data for the chemical composition of the *P. rivinoides* samples; Figure S1: Molecular family of lignoids present in the ethanolic extracts of stems, branches, roots and leaves of *Piper rivinoides*; Figure S2: Molecular family of lignoids present in the ethanolic extracts of stems, branches, roots and leaves of *Piper rivinoides*; Figure S3: Molecular family of lignans present in the ethanolic extracts of stems, branches, roots and leaves of *Piper rivinoides*; Figure S4: Molecular family of lignanamides present in the ethanolic extracts of stems, branches, roots and leaves of *Piper rivinoides*; Figure S5: Molecular family of heterolignans present in the ethanolic extracts of stems, branches, roots and leaves of *Piper rivinoides*; Figure S6: Molecular family of triterpenes present in the ethanolic extracts of stems, branches, roots and leaves of *Piper rivinoides*; Figure S7: Molecular family of carotenoids present in the ethanolic extracts of stems, branches, roots and leaves of *Piper rivinoides*; Figure S8: Molecular families of procyanidins and glycosyl flavanoids present in the ethanolic extracts of stems, branches, roots and leaves of *Piper rivinoides*; Figure S9: Molecular family of amides present in the ethanolic extracts of stems, branches, roots and leaves of *Piper rivinoides*; Figure S10: Molecular family of glycerophosphocholines present in the ethanolic extracts of stems, branches, roots and leaves of *Piper rivinoides*; Figure S11: Mass spectra (ESI, positive mode, MS^2^) and proposed fragmentation pathway for the neolignan rivinoidine A; Figure S12: Mass spectra (ESI, positive mode, MS^2^) and proposed fragmentation pathway for the benzofuran-type neolignan rivinoidine B.

## References

[1] World Health Organization (2024). Global Tuberculosis Report 2024. https://www.who.int/publications/i/item/9789240101531.

[2] Chandra P., Grigsby S.J., Philips J.A. (2022). Immune evasion and provocation by *Mycobacterium tuberculosis*. Nat. Rev. Microbiol..

[3] Huang L., Nazarova E.V., Tan S., Liu Y., Russell D.G. (2018). Growth of *Mycobacterium tuberculosis* in vivo segregates with host macrophage metabolism and ontogeny. J. Exp. Med..

[4] Cohen S.B., Gern B.H., Delahaye J.L., Plumlee C.R., Gerner M.Y., Urdahl K.B. (2020). Alveolar macrophages provide an early *Mycobacterium tuberculosis* niche and initiate dissemination. J. Immunol..

[5] Sun M.R., Xing J.Y., Li X.T., Fang R., Zhang Y., Li Z.L., Song N.N. (2025). Recent advances in research on *Mycobacterium tuberculosis* virulence factors and their role in pathogenesis. J. Microbiol. Immunol. Infect.

[6] Shukla S., Bhardwaj N., Singh A. (2024). Drug resistance in Mycobacterium tuberculosis: An evolutionary perspective and its adaptation to the lung microenvironment. Microbe.

[7] Dong S., Guo X., Han F., He Z., Wang Y. (2022). Emerging role of natural products in cancer immunotherapy. Acta Pharm. Sin. B.

[8] Neto P.T.P.F., Tellis C.J.M., Pimenta F.P. (2022). Novos Derivados de Plantas Medicinais para Tratamento da Tuberculose em Documentos de Patente. Cad. Prospec..

[9] Ramos Y.J.E., Costa-Oliveira C., Fonseca I.C., da Silva Marcelino D., Heggdorne-Araujo M., Lassounskaia E.B., de Lima Moreira D. (2021). *Piper multinodum* C. DC. (Piperaceae) essential oils chemical variation and biological activity against Mycobacterium tuberculosis. J. Med. Plant. Res..

[10] de Luna A.V., Fagundes T.D.S.F., Ramos Y.J., de Araújo M.H., Muzitano M.F., Calixto S.D., Simão T.L.B.V., de Queiroz G.A., Guimarães E.F., Moreira D.D.L. (2024). UHPLC-HRMS/MS Chemical Fingerprinting of the Bioactive Partition from Cultivated *Piper aduncum* L.. Molecules.

[11] Endriyatno N.C. (2022). Molecular Docking of Betel Leaf (*Piper betle* L.) on Protein Dihydrofolate reductase of *Mycobacterium tuberculosis*. Sci. Community Pharm. J..

[12] de Lima Moreira D., de Paiva R.A., Marques A.M., Borges R.M., Barreto A.L.S., da Rocha Curvelo J.A., Cavalcanti J.F., Romanos M.T.V., de Araújo Soares R.M., Kaplan M.A.C. (2016). Bioactive neolignans from the leaves of *Piper rivinoides* Kunth (Piperaceae). Rec. Nat. Prod..

[13] Leal A.L.A.B., Machado A.J.T., Bezerra C.F., Inácio C.E.S., Rocha J.E., Sales D.L., de Freitas T.S., Almeida W.d.O., Amaral W.D., Coutinho H.D.M. (2019). Chemical identification and antimicrobial potential of essential oil of *Piper rivinoides* kunth (BETIS-WHITE). Food Chem. Toxicol..

[14] Felisberto J.E.S., Ramos Y.J.E., de Queiroz G.A., Guimarães E.F., Marques A.E.M., de Lima Moreira D. (2022). Piper rivinoides Kunth: A medicinal plant that preserves bioactive chemical substances in its essential oil throughout the seasons. J. Med. Plants Res..

[15] Bernuci K.Z., Iwanaga C.C., Fernandez-Andrade C.M.M., Lorenzetti F.B., Torres-Santos E.C., Faioes V.D.S., Gonçalves J.E., Amaral W.D., Deschamps C., Cortez D.A.G. (2016). Evaluation of chemical composition and antileishmanial and antituberculosis activities of essential oils of *Piper species*. Molecules.

[16] Fonseca A.C.C.D., de Queiroz L.N., Sales Felisberto J., Jesse Ramos Y., Mesquita Marques A., Wermelinger G.F., Pontes B., Moreira D.d.L., Robbs B.K. (2021). Cytotoxic effect of pure compounds from *Piper rivinoides* Kunth against oral squamous cell carcinoma. Nat. Prod. Res..

[17] Machado T.Q., Felisberto J.R.S., Guimarães E.F., Queiroz G.A.D., Fonseca A.C.C.D., Ramos Y.J., Marques A.M., Moreira D.d.L., Robbs B.K. (2022). Apoptotic effect of β-pinene on oral squamous cell carcinoma as one of the major compounds from essential oil of medicinal plant *Piper rivinoides* Kunth. Nat. Prod. Res..

[18] Ramos Y.J., Gouvêa-Silva J.G., de Brito Machado D., Felisberto J.S., Pereira R.C., Sadgrove N.J., de Lima Moreira D. (2023). Chemophenetic and chemodiversity approaches: New insights on modern study of plant secondary metabolite diversity at different spatiotemporal and organizational scales. Rev. Bras. Farmacog..

[19] Vaistij F.E., Lim E.K., Edwards R., Bowles D.J. (2009). Glycosylation of secondary metabolites and xenobiotics. Plant-Deriv. Nat. Prod. Synth. Funct. Appl..

[20] Müller C., Junker R.R. (2022). Chemical phenotype as important and dynamic niche dimension of plants. New Phytol..

[21] Felisberto J.S., Machado D.B., Assunção J.A., Massau S.A., Queiroz G.A.D., Guimarães E.F., Ramos Y.J., Moreira D.D.L. (2024). Spatio-Temporal Variations of Volatile Metabolites as an Eco-Physiological Response of a Native Species in the Tropical Forest. Plants.

[22] Erb M., Balmer D., De Lange E.S., Von Merey G., Planchamp C., Robert C.A., Röder G., Sobhy I., Zwahlen C., Turlings T.C. (2011). Synergies and trade-offs between insect and pathogen resistance in maize leaves and roots. Plant Cell Environ..

[23] Hazra A., Saha S., Dasgupta N., Kumar R., Sengupta C., Das S. (2021). Ecophysiological traits differentially modulate secondary metabolite accumulation and antioxidant properties of tea plant [*Camellia sinensis* (L.) O. Kuntze]. Sci. Rep..

[24] Hajdari A., Mustafa B., Nebija D., Selimi H., Veselaj Z., Breznica P., Quave C.L., Novak J. (2016). Composition of essential oil from needles and twigs of *Pinus peuce* Griseb. from two national parks of Kosovo. Sci. World J..

[25] Garcia F.P., Lazarin-Bidóia D., Ueda-Nakamura T., Silva S.D.O., Nakamura C.V. (2013). Eupomatenoid-5 Isolated From Leaves of *Piper regnellii* induces Apoptosis in *Leishmania amazonensis*. Evid. Based Complement. Alternat. Med..

[26] Scodro R.B.L., Pires C.T.A., Carrara V.S., Lemos C.O.T., Cardozo-Filho L., Souza V.A., Corrêa A.G., Siqueira V.L.D., Lonardoni M.V.C., Cardoso R.F. (2013). Anti-tuberculosis neolignans from *Piper regnellii*. Phytomedicine.

[27] Felisberto J.S., Marques A.M., de Lima Moreira D. (2021). Development and validation of an analytical method to quantify bioactive neolignans of *Piper rivinoides* Kunth extracts. Rev. Virtual Quim..

[28] Lopes M.A., Ferracioli K.R.C., Siqueira V.L.D., de Lima Scodro R.B., Cortez D.A.G., da Silva R.Z., Cardoso R.F. (2014). In vitro interaction of eupomatenoid-5 from *Piper solmsianum* C. DC. var. *solmsianum* and anti-tuberculosis drugs. Int. J. Tuberc. Lung Dis..

[29] Abbas F., Ke Y., Yu R., Yue Y., Amanullah S., Jahangir M.M., Fan Y. (2017). Volatile terpenoids: Multiple functions, biosynthesis, modulation and manipulation by genetic engineering. Planta.

[30] Badria F.A., Blumenberg M. (2022). Phenolic Compounds: Chemistry, Synthesis, Diversity, Industrial, Pharmaceutical and Unconventional Therapeutic Applications.

[31] Bai J., Chen H., Fang Z.F., Yu S.S., Ma S.G., Li Y., Qu J., Xu S., Ren J.H., Lu H.N. (2012). Sesquiterpenes and neolignans from the roots of *Illicium dunnianum*. J. Asian Nat. Prod. Res..

[32] Gottlieb O.R. (1998). Lignoids from Amazonian plants: Biological and chemical investigations. Acta Amaz..

[33] Cabral M.M.O., Azambuja P., Gottlieb O.R., Garcia E.S. (2000). Effects of some lignans and neolignans on the development and excretion of *Rhodnius prolixus*. Fitoterapia.

[34] Teponno R.B., Kusari S., Spiteller M. (2016). Recent advances in research on lignans and neolignans. Nat. Prod. Rep..

[35] Puglielli G., Laanisto L., Gori A., Cardoso A.A. (2023). Adaptations of Woody Plants to Multiple Abiotic Stressors: Where Do We Stand?. Flora.

[36] Góral I., Jurek I., Wojciechowski K. (2018). How does the surface activity of soapwort (*Saponaria officinalis* L.) extracts depend on the plant organ?. J. Surfactants Deterg..

[37] Gershenzon J., Ullah C. (2022). Plants protect themselves from herbivores by optimizing the distribution of chemical defenses. Proc. Natl. Acad. Sci. USA.

[38] Xu Y., Liang B., Kong C., Sun Z. (2021). Traditional medicinal plants as a source of antituberculosis drugs: A system review. Biomed Res. Int..

[39] Conradie F., Diacon A.H., Ngubane N., Howell P., Everitt D., Crook A.M., Mendel C.M., Egizi E., Moreira J., Spigelman M. (2020). Treatment of Extensively Drug-Resistant Pulmonary Tuberculosis. N. Engl. J. Med..

[40] Chen J.J., Wang T.Y., Hwang T.L. (2008). Neolignans, a coumarinolignan, lignan derivatives, and a chromene: Anti-inflammatory constituents from Zanthoxylum avicennae. J. Nat. Prod..

[41] Elsaidi H.R., Barreda D.R., Cairo C.W., Lowary T.L. (2013). Mycobacterial Phenolic Glycolipids with a Simplified Lipid Aglycone Modulate Cytokine Levels through Toll-like Receptor 2. ChemBioChem.

[42] Shaku M., Ealand C., Kana B.D. (2020). Cell surface biosynthesis and remodeling pathways in mycobacteria reveal new drug targets. Front. Cell. Infect. Microbiol..

[43] Abrahams K.A., Besra G.S. (2018). Mycobacterial cell wall biosynthesis: A multifaceted antibiotic target. Parasitology.

[44] Almeida F.M., Ventura T.L., Amaral E.P., Ribeiro S.C., Calixto S.D., Manhaes M.R., Rezende A.L., Souzal G.S., de Carvalho I.S., Lasunskaia E.B. (2017). Hypervirulent *Mycobacterium tuberculosis* strain triggers necrotic lung pathology associated with enhanced recruitment of neutrophils in resistant C57BL/6 mice. PLoS ONE.

[45] Dokrungkoon T., Tulyaprawat O., Suwannakarn K., Ngamskulrungroj P. (2023). In vitro modeling of isoniazid resistance mechanisms in *Mycobacterium tuberculosis* H37Rv. Front. Microbiol..

[46] Oppong Y.E., Phelan J., Perdigão J., Machado D., Miranda A., Portugal I., Hibberd M.L. (2019). Genome-wide analysis of *Mycobacterium tuberculosis* polymorphisms reveals lineage-specific associations with drug resistance. BMC Genom..

[47] Maitra A., Munshi T., Healy J., Martin L.T., Vollmer W., Keep N.H., Bhakta S. (2019). Cell wall peptidoglycan in *Mycobacterium tuberculosis*: An Achilles’ heel for the TB-causing pathogen. FEMS Microbiol. Rev..

[48] Zodape G.V., Dharmashale S.N., Gaikwad V.S. (2021). Effect of *Piper nigrum* (Linn.) seeds extract and second line anti-tuberculosis drugs on a few *Mycobacterium tuberculosis* strains. J. Appl. Nat. Sci..

[49] Johann S., Cota B.B., Souza-Fagundes E.M., Pizzolatti M.G., Resende M.A., Zani C.L. (2009). Antifungal activities of compounds isolated from *Piper abutiloides* Kunth. Mycoses.

[50] Scodro R.B.D.L., Espelho S.C., Pires C.T.A., Garcia V.A.D.S., Cardozo-Filho L., Cortez L.E., Cortez D.A. (2015). A new benzoic acid derivative from *Piper diospyrifolium* and its anti-Mycobacterium tuberculosis activity. Phytochem. Lett..

[51] Fernandez C.M., Baldin V.P., Ieque A.L., Bernuci K.Z., Almeida R.T., Valone L.M., Dias Filho B.P. (2019). *Anti-Mycobacterium tuberculosis* activity of dichloromethane extract of *Piper corcovadensis* (Miq.) C. DC. roots and isolated compounds. Ind. Crops Prod..

[52] Apers S., Vlietinck A., Pieters L. (2003). Lignans and neolignans as lead compounds. Phytochem. Rev..

[53] Mazlun M.H., Sabran S.F., Mohamed M., Abu Bakar M.F., Abdullah Z. (2019). Phenolic compounds as promising drug candidates in tuberculosis therapy. Molecules.

[54] Ghiraldi-Lopes L.D., Campanerut-Sá P.A., Meneguello J.E., Seixas F.A., Lopes-Ortiz M.A., Scodro R.B., Cardoso R.F. (2017). Proteomic profile of *Mycobacterium tuberculosis* after eupomatenoid-5 induction reveals potential drug targets. Future Microbiol..

[55] Longato G.B., Rizzo L.Y., de Oliveira Sousa I.M., Tinti S.V., Possenti A., Figueira G.M., de Carvalho J.E. (2011). In vitro and in vivo anticancer activity of extracts, fractions, and eupomatenoid-5 obtained from *Piper regnellii* leaves. Planta Med..

[56] Pelizzaro-Rocha K.J., Veiga-Santos P., Lazarin-Bidóia D., Ueda-Nakamura T., Dias Filho B.P., Ximenes V.F., Silva O.S., Nakamura C.V. (2011). Trypanocidal action of eupomatenoid-5 is related to mitochondrion dysfunction and oxidative damage in *Trypanosoma cruzi*. Microbes Infect..

[57] Lazarin-Bidóia D., Desoti V.C., Ueda-Nakamura T., Dias Filho B.P., Nakamura C.V., Silva S.O. (2013). Further evidence of the trypanocidal action of eupomatenoid-5: Confirmation of involvement of reactive oxygen species and mitochondria owing to a reduction in trypanothione reductase activity. Free Radic. Biol. Med..

[58] Luize P.S., Ueda-Nakamura T., Dias Filho B.P., Cortez D.A.G., Nakamura C.V. (2006). Activity of Neolignans Isolated from *Piper regnellii* (M IQ.) C. DC. var. *pallescens* (C. DC.) Y UNCK against *Trypanosoma cruzi*. Biol. Pharm. Bull..

[59] Pessini G.L., Dias Filho B.P., Nakamura C.V., Cortez D.A.G. (2003). Antibacterial activity of extracts and neolignans from *Piper regnellii* (Miq.) C. DC. var. *pallescens* (C. DC.). Yunck. Mem. Inst. Oswaldo Cruz..

[60] Lemos C.O.T., Svidzinsk T.I.E., Baeza L.C., Miranda N., Nakamura C.V., Cortez D.A.G., Cardozo-Filho L., Cabral V.F. (2013). Evaluation of antifungal activity of extracts of *Piper regnellii* obtained by supercritical fluid extraction. Nat. Prod. Res..

[61] Silva J.G.D., Souza I.A., Higino J.S., Siqueira-Junior J.P., Pereira J.V., Pereira M.D.S.V. (2007). Antimicrobial Activity of *Anacardium occidentale* Linn. Extract Against Multidrug-Resistant *Staphylococcus aureus* Strains. Rev. Bras. Farmacogn..

[62] Bohatch Júnior M.S., Esmerino L.A., Zanoni da Silva R., Volpato A.M. (2016). Effects of the Antimicrobial Activity of the Crude Ethanolic Extract of *Piper solmsianum* and Equisetum arvense. Electron. J. Pharm..

[63] Ortiz S., Lecsö-Bornet M., Bonnal C., Houze S., Michel S., Grougnet R., Boutefnouchet S. (2019). Bioguided identification of triterpenoids and neolignans as bioactive compounds from anti-infectious medicinal plants of the Taira Atacama’s community (Calama, Chile). J. Ethnopharmacol..

[64] Dall’Igna D.M., Steil A.A., da Silva R.Z., Cechinel Filho V., Cruz A.B. (2019). The effect of conocarpan on susceptibility of *Candida albicans* to phagocytosis and digestion by macrophages. Braz. J. Biol. Sci..

[65] Marques A., Paiva R., Kaplan M.A., Moreira D., Barreto A.L., Curvelo A. (2014). Bioactive neolignans from *Piper rivinoides* Kunth (Piperaceae). Planta Med..

[66] MassBank of North America (MoNA). https://mona.fiehnlab.ucdavis.edu/.

[67] Zálešák F., Bon D.J.Y.D., Pospíšil J. (2019). Lignans and neolignans: Plant secondary metabolites as a reservoir of biologically active substances. Pharmacol. Res..

[68] Pan L.L., Wang X.L., Luo X.L., Liu S.Y., Xu P., Hu J.F., Liu X.H. (2017). Boehmenan, a lignan from the Chinese medicinal plant *Clematis armandii*, inhibits A431 cell growth via blocking p70S6/S6 kinase pathway. Integr. Cancer Ther..

[69] Quynh D.T.D., Vu T.N., Mai H.D.T., Ngan T., Cuông P.V., Thạch T.D., Huong D.T.M., Thái T.L., Hieu T., Thao T.T. (2018). Chemical constituents of MeOH extract from the fruits of *Macaranga sampsonii*. Vietnam J. Chem..

[70] Rudiyansyah R., Lambert L.K., Garson M.J. (2010). Lignans and triterpenes from the bark of *Durio carinatus* and *Durio oxleyanus*. J. Nat. Prod..

[71] Wu Y., Zheng C., Deng X.H., Qin L.P. (2013). Two new bis-alkaloids from the aerial part of *Piper flaviflorum*. Helv. Chim. Acta.

[72] Chandra P., Pandey R., Srivastva M., Rameshkumar K.B., Kumar B. (2015). Quantitative determination of chemical constituents of *Piper* spp. using UPLC--ESI--MS/MS. Ind. Crops Prod..

[73] Arunachalam K., Damazo A.S., Macho A., da Silva Lima J.C., Pavan E., de Freitas Figueiredo F., Oliveira D.M., Filho V.C., Wagner T.M., de Oliveira Martins D.T. (2020). *Piper umbellatum* L. (Piperaceae): Phytochemical profiles of the hydroethanolic leaf extract and intestinal anti-inflammatory mechanisms on 2,4,6-trinitrobenzene sulfonic acid-induced ulcerative colitis in rats. J. Ethnopharmacol..

[74] Rivera S., Vilaró F., Canela-Garayoa R. (2011). Determination of carotenoids by liquid chromatography/mass spectrometry: Effect of several dopants. Anal. Bioanal. Chem..

[75] Demarque D.P., Crotti A.E., Vessecchi R., Lopes J.L., Lopes N.P. (2016). Fragmentation reactions using electrospray ionization mass spectrometry: An important tool for the structural elucidation and characterization of synthetic and natural products. Nat. Prod. Rep..

[76] Parmar V.S., Jain S.C., Bisht K.S., Jain R., Taneja P., Jha A., Tyagi D., Prasad A.K., Wengel J., Olsen C.E. (1997). Phytochemistry of the genus *Piper*. Phytochemistry.

[77] Moreira D.L., Guimarães E.F., Kaplan M.C. (2000). A *C*-glucosylflavone from leaves of *Piper lhotzkyanum*. Phytochemistry.

[78] Ticona J.C., Bilbao-Ramos P., Amesty Á., Flores N., Dea-Ayuela M.A., Bazzocchi I.L., Jiménez I.A. (2022). Flavonoids from *Piper* species as promising antiprotozoal agents against *Giardia intestinalis*: Structure-activity relationship and drug-likeness studies. Pharmaceuticals.

[79] Nongmai C., Kanokmedhakul K., Promgool T., Paluka J., Suwanphakdee C., Kanokmedhakul S. (2022). Chemical constituents and antibacterial activity from the stems and leaves of *Piper wallichii*. J. Asian Nat. Prod. Res..

[80] Olalere O.A., Abdurahman N.H., bin Mohd Yunus R., Alara O.R., Ahmad M.M. (2019). Mineral element determination and phenolic compounds profiling of oleoresin extracts using an accurate mass LC-MS-QTOF and ICP-MS. J. King Saud Univ. Sci..

[81] Zhang J., Guan S., Sun J., Liu T., Chen P., Feng R., Chen X., Wu W., Yang M., Guo D.-A. (2015). Characterization and profiling of phenolic amides from *Cortex Lycii* by ultra-high performance liquid chromatography coupled with LTQ-Orbitrap mass spectrometry. Anal. Bioanal. Chem..

[82] Van der Rest B., Boisson A.M., Gout E., Bligny R., Douce R. (2002). Glycerophosphocholine metabolism in higher plant cells. Evidence of a new glyceryl-phosphodiester phosphodiesterase. Plant Physiol..

[83] Garrett T.J., Merves M., Yost R.A. (2011). Characterization of protonated phospholipids as fragile ions in quadrupole ion trap mass spectrometry. Int. J. Mass Spectrom..

[84] Yan S., Liu Q., Li W., Yan J., Fernie A.R. (2022). Raffinose family oligosaccharides: Crucial regulators of plant development and stress responses. Crit. Rev. Plant Sci..

[85] Azmir J., Zaidul I.S.M., Rahman M.M., Sharif K.M., Mohamed A., Sahena F., Jahurul M.H.A., Ghafoor K., Norulaini N.A.N., Omar A.K.M. (2013). Techniques for extraction of bioactive compounds from plant materials: A review. J. Food Eng..

[86] Da Cruz A.F.G., Reis A.C.C., Sousa J.A.C., Vaz L.B.A., de Mello Silva B., de Brito Magalhães C.L., Kohlhoff M., de Oliveira A.B., Brandão G.C. (2022). High-resolution mass spectrometry identification and characterization of flavonoids from Fridericia chica leaves extract with anti-arbovirus activity. Molecules.

[87] Wang M., Carver J.J., Phelan V.V., Sanchez L.M., Garg N., Peng Y., Nguyen D.D., Watrous J., Kapono C.A., Bandeira N. (2016). Sharing and community curation of mass spectrometry data with Global Natural Products Social Molecular Networking. Nat. Biotechnol..

[88] Zhang N., Jing T., Zhao M., Jin J., Xu M., Chen Y., Zhang S., Xiaochun W., Schwab W., Song C. (2019). Untargeted metabolomics coupled with chemometrics analysis reveals potential non-volatile markers during oolong tea shaking. Food Res. Intern..

[89] Ribeiro L.P. (2014). Exploring Genetic Biodiversity: Secondary Metabolites from Neotropical Annonaceae as a Potential Source of New Pesticides. Ph.D. Thesis.

