# Supplementary material for: Organ-Specific Distribution of Antimycobacterial Neolignans in Piper rivinoides and UHPLC-HRMS/MS Analysis of Its Extracts
Source: Molecules. 2025 Dec 6;30(24):4682. doi: 10.3390/molecules30244682 (PMC12735719; doi:10.3390/molecules30244682)
Supplement: Supplementary file 1 [file molecules-30-04682-s001.zip › molecules-3934156-supplementary.pdf]

Supplementary Materials

Table S1 - UHPLC-HRMS-MS data for the chemical composition of the *P. rivinoides* samples*

Compound	Molecular Formula	Rt (min)	Adduct ion	Experimental *m/z*	Theoretical *m/z*	Error (ppm)	Main Ions of MS2 spectrum	Shared peaks	Annotation Type
**Lignoids**									
conocarpan (**1**)	C18H18O2	26.3	$[M+H]^+$	267.1389	267.1385	1.5	223.07; 157.06; 145.06; 131.04; 117.07; 115.05; 107.04; 105.07	6	GNPS library
parakmerin A (**2**)	C19H20O3	26.6	$[M+H]^+$	297.1486	297.14906	-1.5	145.06; 137.06; 128.06; 117.07; 115.05; 103.05	-	Manual inspection
1,2-benzenediol, 4-(2,3-dihydro-3-methyl-5-(1-propen-1-yl-2-benzofuranyl) (**3**)	C18H18O3	24.2	$[M+H]^+$	283.1339	283.13341	1.7	145.06; 123.04; 115.05; 103.05	-	Manual library
2,3-dihydro-2-(4-hydroxy-3-methoxyphenyl)-3-methyl-5-benzofurancarboxaldehyde (**4**)	C17H16O4	19.8	$[M+H]^+$	285.1136	285.11268	3.2	128.06; 107.05; 105.06; 103.05	-	Manual inspection
decurrenal (**5**)	C16H14O3	19.5	$[M+H]^+$	255.1019	255.10211	-0.8	165.07; 131.04; 115.0552; 107.05; 105.07; 103.05	-	Manual inspection
2,3-dihydro-2-(4-hydroxyphenyl)-5-(1-propen-1-yl)-3-benzofuranmethanol (**6**)	C18H18O3	15.6	$[M+H]^+$	283.1326	283.13341	-2.9	149.06; 133.06; 131.04; 128.05; 107.04; 105.06	-	Manual inspection
rivinoidine A (**7**)	C18H20NO2	15.7	$[M+H]^+$	282.1507	282.1494	4.6	145.06; 133.06; 115.06; 107.04; 105.06; 103.05	-	Manual inspection
eupomatenoid 5 (**8**)	C19H18O3	28.5	$[M+Na]^+$	317.1142	317.11536	-3.7	No fragments	-	Manual inspection
			$[M+H]^+$	295.1331	295.13341	-1.1	237.09; 219.08; 207.08; 191.08; 179.08; 178.07; 165.07; 128.06; 115.05	4	GNPS library
eupomatenoid 3 (**9**)	C19H16O3	24.3	$[M+H]^+$	293.1191	293.11776	4.6	247.07; 219.08; 207.08; 191.08; 179.08; 178.07; 165.07; 135.04; 115.05	-	Manual inspection
eupomatenoid 6 (**10**)	C18H16O2	28.1	$[M+H]^+$	265.1236	265.12285	2.8	249.09; 223.07; 207.08; 194.07; 181.06; 178.0779; 165.0725; 141.07; 128.06; 119.05; 115.05; 107.04	-	Manual inspection
eupomatenoid 15 (**11**)	C19H18O2	24.9	$[M+H]^+$	279.1391	279.1385	2.1	249.09; 221.10; 207.08; 179.08; 128.06; 118.04	-	Manual inspection
5-hydroxy-eupomatenoid 6 (**12**)	C18H16O3	25.7	$[M+H]^+$	281.1186	281.11776	0.9	265.09; 239.07; 219.08; 202.08; 191.08; 190.07; 179.08; 178.08; 165.07; 141.07; 128.06; 115.05	-	Manual inspection
9-hydroxy-eupomatenoid 5 (**13**)	C19H19O4	24.3	$[M+H]^+$	311.1281	311.12833	-0.7	278.09; 265.08; 251.06; 220.09; 207.08; 194.07; 192.09; 191.08; 179.08; 178.08; 165.07; 128.06; 115.05	-	Manual inspection
			$[2M+Na]^+$	643.2307	643.23078	-0.1	333.1098	-	Manual inspection
rivinoidine B (**14**)	C19H19NO3	17.0	$[M+H]^+$	310.1448	310.14431	1.6	253.09; 239.07; 238.06; 211.07; 207.08; 166.07; 165.07	-	Manual inspection

9-hydroxy-parakmerin A (**15**)	C19H20O4	19.3	$[2M+Na]^+$	647.2600	647.26208	-3.2	335.1251	-	Manual inspection
			$[M+Na]^+$	335.1253	335.12592	-1.9	No fragments	-	Manual inspection
eupomatenoid A (**16**)	C40H40O11	20.3	$[M+Na]^+$	719.2437	719.24683	-4.4	393.12; 349.10; 309.11	-	Manual inspection
eupomatenoid B (**17**)	C38H36O10	20.4	$[M+Na]^+$	675.2194	675.22061	-1.8	349.10; 309.11	-	Manual inspection
eupomatenoid C (**18**)	C38H38O9	21.0	$[M+Na]^+$	661.2400	661.24135	-2.0	349.10; 335.12	-	Manual inspection
eupomatenoid D (**19**)	C38H36O10	23.2	$[M+Na]^+$	675.2191	675.22061	-2.2	349.10; 151.04	-	Manual inspection
**Lignanamides**									
flavifloramide B (**20**)	C38H40N2O10	16.1	$[M+H]^+$	685.2738	685.27612	-3.4	520.19; 383.11; 357.13; 352.09; 351.08; 325.10; 265.08; 231.06; 121.06	13	GNPS library
flavifloramide A (**21**)	C37H38N2O10	15.4	$[M+H]^+$	671.2582	671.26047	-3.4	506.16; 369.09; 337.07; 309.07; 231.06; 217.0459; 121.06	-	Manual inspection
melongenamide A (**22**)	C37H38N2O9	16.3	$[M+H]^+$	655.2652	655.26555	-0.5	490.20; 353.10; 322.07; 321.07; 295.09; 263.07; 231.06; 121.06	-	Manual inspection
cannabisin D (**23**)	C36H36N2O8	18.4	$[M+H]^+$	625.2534	625.25499	-2.5	351.08; 325.10; 307.09; 293.08; 265.08; 201.05; 121.06	-	Manual inspection
7''-hydroxy-flavifloramide A (**24**)	C37H38N2O11	14.3	$[M+H]^+$	687.2541	687.25538	-1.9	370.09; 369.09; 337.06; 309.07; 231.06; 217.05; 137.06	-	Manual inspection
**Heterolignans**									
2-[3-[2-(4-hydroxy-3-methoxyphenyl)-3-(hydroxymethyl)-7-methoxy-2,3-dihydro-1-benzofuran-5-yl]prop-2-enoyl]amino]pentanedioic acid (**25**)	C25H27NO10	13.4	$[M+H]^+$	502.1680	502.1713	-6.6	104.10; 137.06; 249.09; 262.05; 277.09; 290.06; 305.08; 322.09	8	GNPS library
2-[3-[2-(4-hydroxy-3,5-dimethoxyphenyl)-3-(hydroxymethyl)-7-methoxy-2,3-dihydro-1-benzofuran-5-yl]prop-2-enoyl]amino]pentanedioic acid (**26**)	C26H29NO11	13.3	$[M+H]^+$	532.1800	532.1818	-3.4	107.05; 121.05; 167.07; 177.05; 191.07; 201.06; 213.05; 264.08; 292.08; 307.10; 320.07; 335.09; 352.08	-	Manual inspection
**Triterpenoids**									
ursolic acid (**27**)	C30H48O3	31.8	$[M+H]^+$	439.3566	439.3576	-2.3	203.18; 189.16; 175.15; 149.13; 147.11; 135.11; 133.10; 121.10; 119.08; 107.08	8	GNPS library
α-amyrenone (**28**)	C30H48O	37	$[M+H]^+$	425.3793	425.37834	2.3	173.13; 149.13; 147.11; 133.09; 121.09; 109.10; 107.08	-	Manual inspection
ursonic acid(**29**)	C30H46O3	33.2	$[M+H]^+$	455.3505	455.35252	-4.4	171.11; 159.11; 147.11; 119.08; 109.10; 107.08	-	Manual inspection
5,6-dehydro-ursonic acid (**30**)	C30H44O3	28.7	$[M+H]^+$	453.3373	453.33687	0.9	173.13; 159.12; 145.1044; 133.10; 131.09; 107.08; 105.06	-	Manual inspection
2-hydroxy-5,6-dehydro-ursonic acid (**31**)	C30H44O4	28.3	$[M+H]^+$	469.3321	469.33178	0.7	197.13; 185.13; 183.12; 173.13; 159.11; 147.11; 145.10; 133.10; 131.08; 121.10; 119.08; 105.07	-	Manual inspection
**Carotenoids**									
β-carotene (**32**)	C40H57	44.2	M^+	536.4347	536.4382	-6.5	496.2903; 211.1494; 197.1305; 185.1328;	7	GNPS library

							183.1166; 171.1153; 157.1025; 146.1172; 145.1006; 133.1017; 121.1008; 119.086; 105.0699		
lutein (**33**)	C40H56O2	36.8	M^+	568.4259	568.42803	-3.7	237.16; 223.14; 209.13; 197.13; 185.13; 173.13; 159.11; 157.09; 145.10; 133.10; 131.08; 119.08; 105.07	-	Manual inspection
echinenone (**34**)	C40H55O	36.8	$[M+H]^+$	551.4217	551.42529	-6.5	256.26; 209.13; 197.13; 183.11; 159.11; 145.10; 133.10; 119.08; 105.07	-	Manual inspection
antheraxanthin (**35**)	C40H56O3	38.9	$[M+H]^+$	585.4278	585.43077	-5.1	309.32; 281.29; 211.14; 197.13; 183.11; 173.13; 171.11; 159.11; 157.10; 145.10; 133.10; 121.1; 119.08; 105.07	-	Manual inspection
zeaxanthin (**36**)	C40H56O2	44.9	$[M+H]^+$	569.4357	569.43585	-0.3	471.36; 266.19, 241.20; 209.14; 197.13; 195.11; 185.13; 183.11; 175.14; 159.11; 145.09; 133.09; 119.08; 105.06	-	Manual inspection
**Flavonoids**									
quercetin 3,7-dirhamnoside (**37**)	C27H30O15	11.3	$[M+H]^+$	595.1639	595.16629	-4.0	303.05; 287.05	6	GNPS library
rutin (**38**)	C27H30O16	10.4	$[M+H]^+$	611.1625	611.1612	2.1	303.05	4	GNPS library
ombuin-3-*O*-rutinoside (**39**)	C29H34O16	25.4	$[M+H]^+$	639.199	639.19251	5.5	331.09	-	Manual inspection
genkwanin (**40**)	C16H12O5	20.6	$[M+H]^+$	285.0760	285.07629	-0.7	224.05; 213.05; 197.05; 187.04; 167.04; 124.02	9	GNPS library
catechin (**41**)	C15H14O6	9.3	$[M+H]^+$	291.0864	291.08686	-1.6	161.06; 147.04; 139.03; 123.04; 119.04	5	GNPS library
procyanidin B2 (**42**)	C30H26O12	8.8	$[M+H]^+$	579.1496	579.15025	-1.1	409.08; 287.05; 271.06; 163.03; 139.03; 127.03; 123.04	11	GNPS library
procyanidin C1 (**43**)	C45H38O18	10	$[M+H]^+$	867.2129	867.21363	-0.8	545.10; 409.09; 407.07; 289.07; 271.06; 247.06; 245.04; 163.04; 139.04; 127.04	-	Manual inspection
**Amides**									
moupinamide (**44**)	C18H19NO4	14.4	$[M+H]^+$	314.1390	314.13923	-0.7	177.05; 149.06; 145.03; 121.06; 117.03; 103.05	6	GNPS library
N-trans-sinapoyltyramine (**45**)	C19H21NO5	14.6	$[M+H]^+$	344.1490	344.14979	-2.3	175.04; 147,04; 121.06; 119.05; 103.05	-	Manual inspection
**Glycerophosphocholines**									
PC(0:0/18:1) (**46**)	C26H52NO7P	27.9	$[M+H]^+$	522.3570	522.35596	2.0	125.00; 184.07	5	GNPS library
PC(0:0/18:0) (**47**)	C26H54NO7P	30.8	$[M+H]^+$	524.3700	524.37161	-3.1	104.11; 125.00; 184.42	7	GNPS library
PC(18:2/0:0) (**48**)	C26H50NO7P	26.0	$[M+H]^+$	520.3410	520.34031	1.3	104.11; 125.00; 184.07	5	GNPS library
PC(18:1/18:1) (**49**)	C44H84NO8P	37.5	$[M+H]^+$	786.5920	786.5987	-3.3	146.98; 184.07; 242.93	6	GNPS library
PC(16:0/18:1) (**50**)	C42H82NO8P	41.4	$[M+Na]^+$	782.5670	782.56757	-0.7	125.00; 146.98; 184.07	9	GNPS library
PC(16:1/18:2) (**51**)	C42H78NO8P	41.7	$[M+H]^+$	756.5550	756.55433	0.9	102.12; 125.00; 184.07	6	GNPS library

PC(0:0/16:0) (**52**)	$C_{24}H_{50}NO_7P$	27.7	$[M+H]^+$	496.3400	496.34031	-0.6	98.98; 104.11; 125.00; 184.07	5	GNPS library
PC(18:3/18:3) (**53**)	$C_{44}H_{76}NO_8P$	41.8	$[M+H]^+$	778.5300	778.53867	-6.1	146.98; 184.07; 573.49; 595.47; 719.49	6	GNPS library
PC(16:0/20:5) (**54**)	$C_{44}H_{78}NO_8P$	42.8	$[M+H]^+$	780.55	780.55433	-5.5	125.00; 146.98; 184.07; 575.50; 597.48	12	GNPS library
PC(18:3/0:0) (**55**)	$C_{26}H_{48}NO_7P$	24.8	$[M+H]^+$	518.3260	518.3246	2.7	104.11; 125.00; 184.07	4	GNPS library
PC(16:0/18:2) (**56**)	$C_{42}H_{80}NO_8P$	40.0	$[M+H]^+$	758.565	758.5699	-6.5	102.13; 184.07; 283.26	9	GNPS library
**Saccharides**									
stachyose (**57**)	$C_{24}H_{42}O_{21}$	56.7	$[M+Na]^+$	689.2081	689.21162	-5.1	112.90; 365.11; 527.16	9	GNPS library
raffinose (**58**)	$C_{18}H_{32}O_{16}$	62.3	$[M+Na]^+$	527.1560	527.1588	-5.3	102.09; 152.07; 203.05; 365.10	4	GNPS library

* Compounds are listed in accordance with chemical classes and not primarily by retention time.

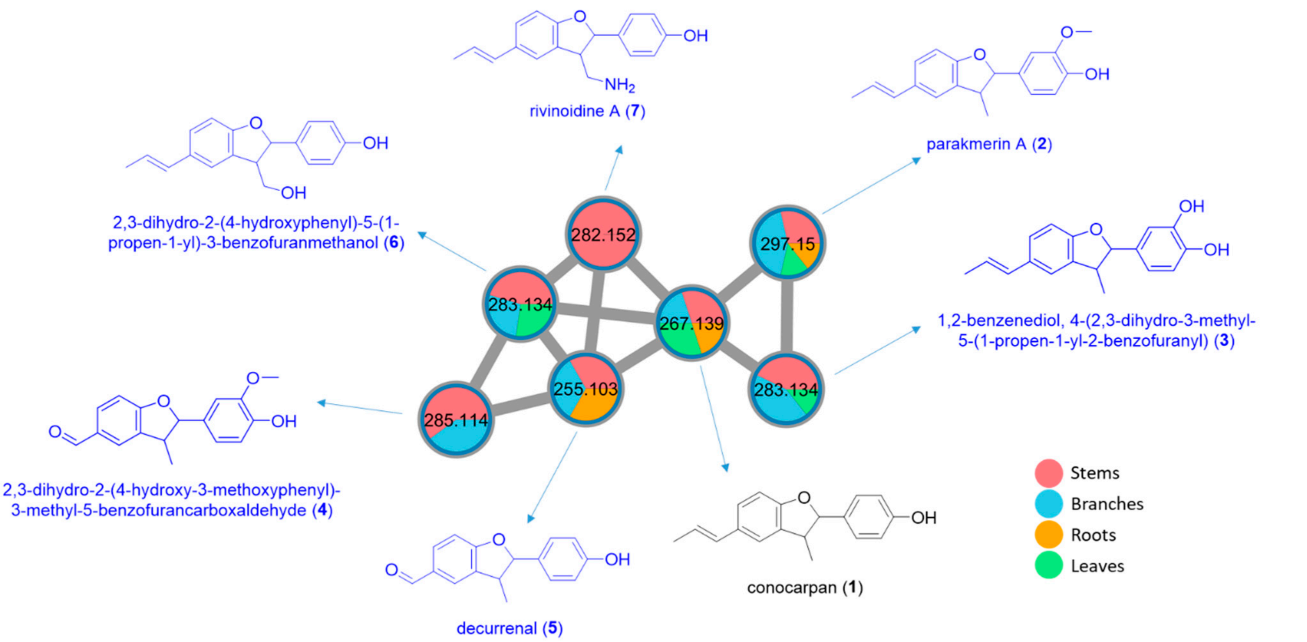

Figure S1 - Molecular family of lignoids present in the ethanolic extracts of stems, branches, roots and leaves of *Piper rivinoides*. In blue, substances annotated through manual inspection of the data, and in black, substances annotated by comparison with the GNPS library. All substances were proposed based on fragmentations that gave rise to characteristic ions and fragmentation profiles.

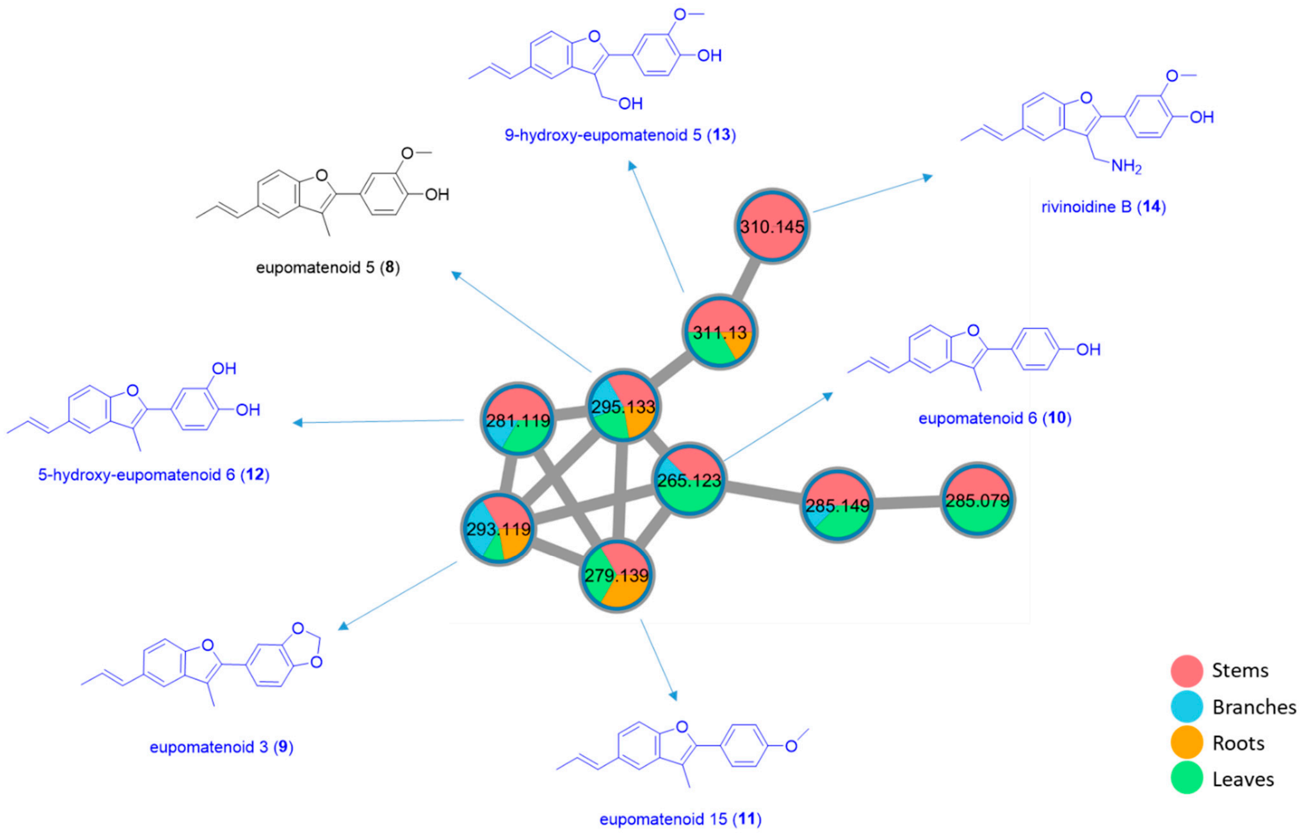

Figure S2 - Molecular family of lignoids present in the ethanolic extracts of stems, branches, roots and leaves of *Piper rivinoides*. In blue, substances annotated through manual inspection of the data, and in black, substances annotated by comparison with the GNPS library. All substances were proposed based on fragmentations that gave rise to characteristic ions and fragmentation profiles.

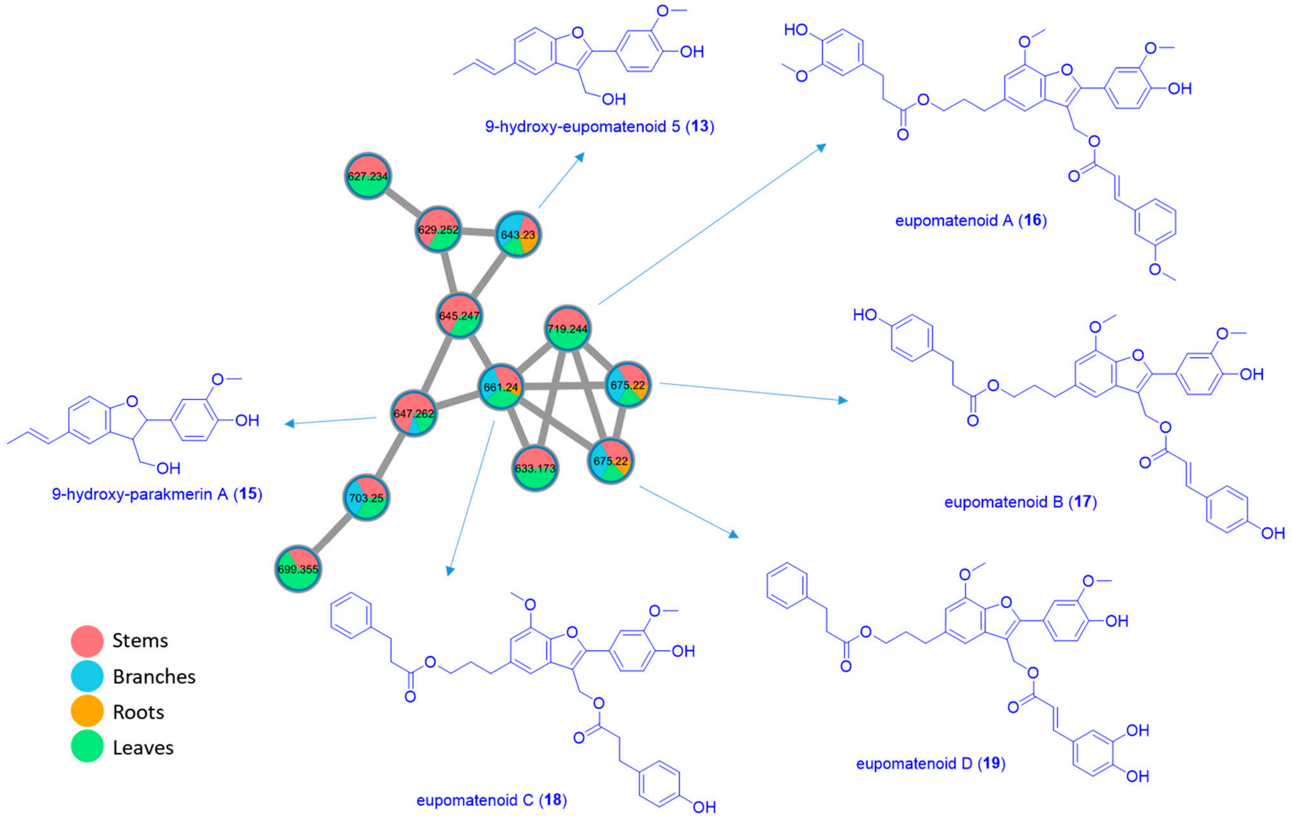

Figure S3 - Molecular family of lignans present in the ethanolic extracts of stems, branches, roots and leaves of *Piper rivinoides*. In blue, substances annotated through manual inspection of the data. All substances were proposed based on fragmentations that gave rise to characteristic ions and fragmentation profiles.

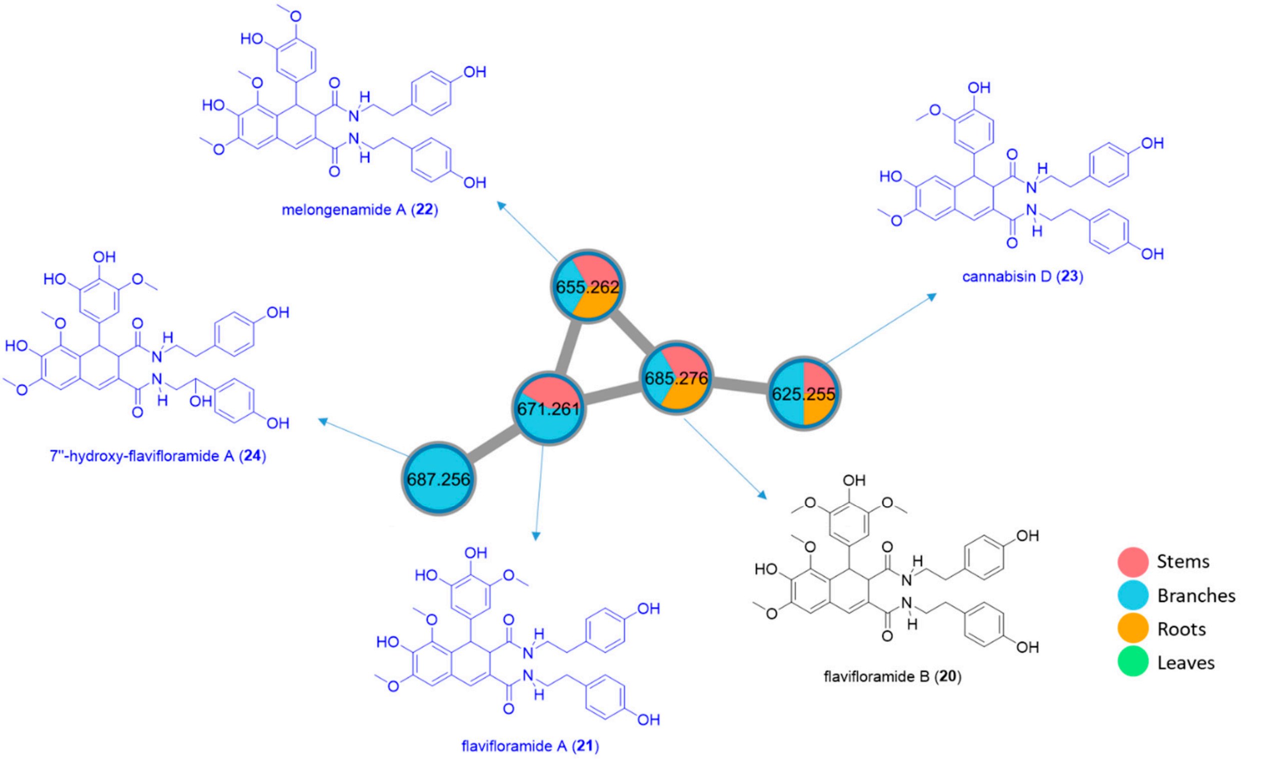

Figure S4 - Molecular family of lignanamides present in the ethanolic extracts of stems, branches, roots and leaves of *Piper rivinoides*. In blue, substances annotated through manual inspection of the data, and in black, substances annotated by comparison with the GNPS library. All substances were proposed based on fragmentations that gave rise to characteristic ions and fragmentation profiles.

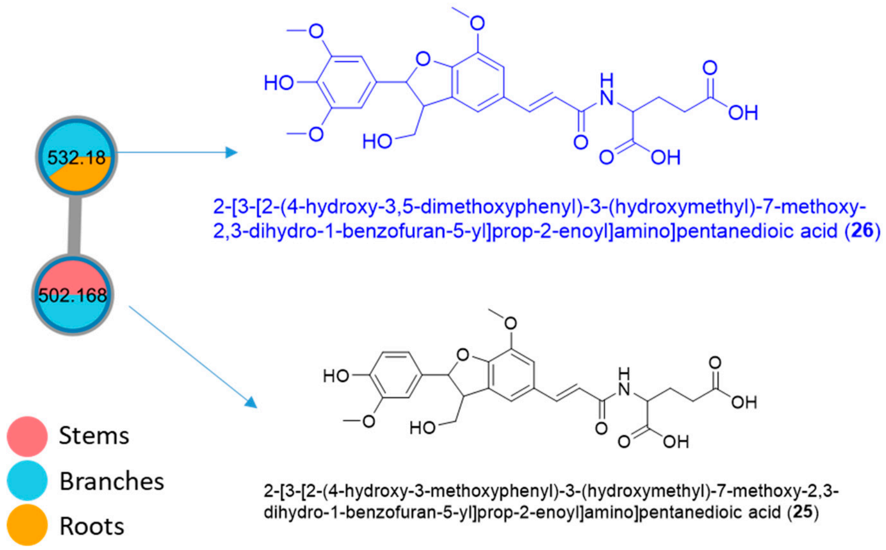

Figure S5 - Molecular family of heterolignans present in the ethanolic extracts of stems, branches, roots and leaves of *Piper rivinoides*. In blue, substances annotated through manual inspection of the data, and in black, substances annotated by comparison with the GNPS library. All substances were proposed based on fragmentations that gave rise to characteristic ions and fragmentation profiles.

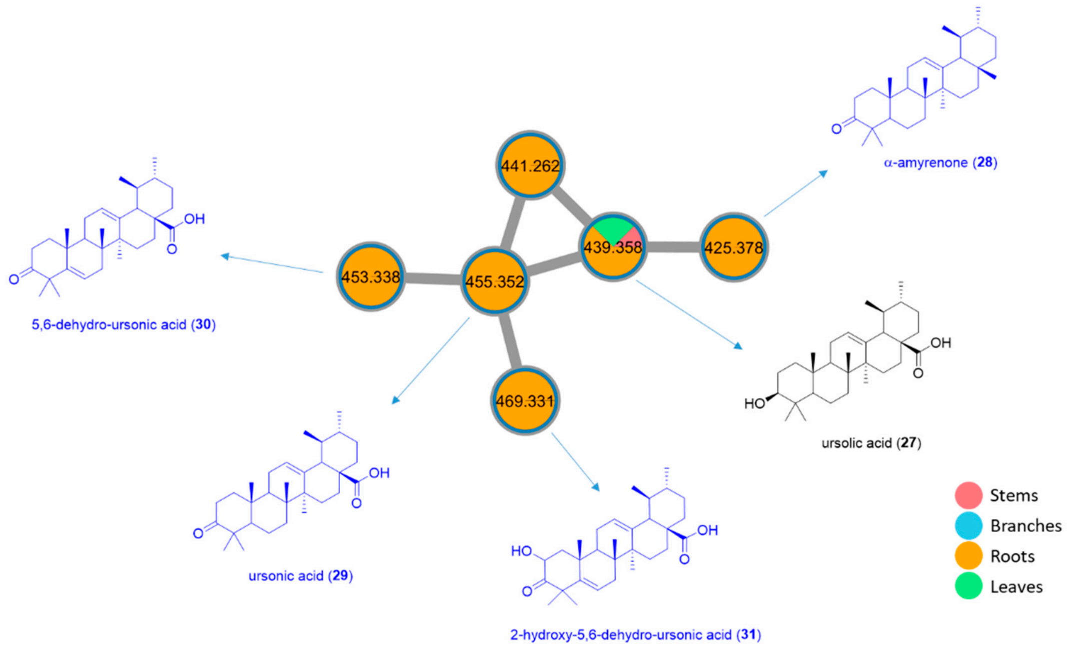

Figure S6 - Molecular family of triterpenes present in the ethanolic extracts of stems, branches, roots and leaves of *Piper rivinoides*. In blue, substances annotated through manual inspection of the data, and in black, substances annotated by comparison with the GNPS library. All substances were proposed based on fragmentations that gave rise to characteristic ions and fragmentation profiles.

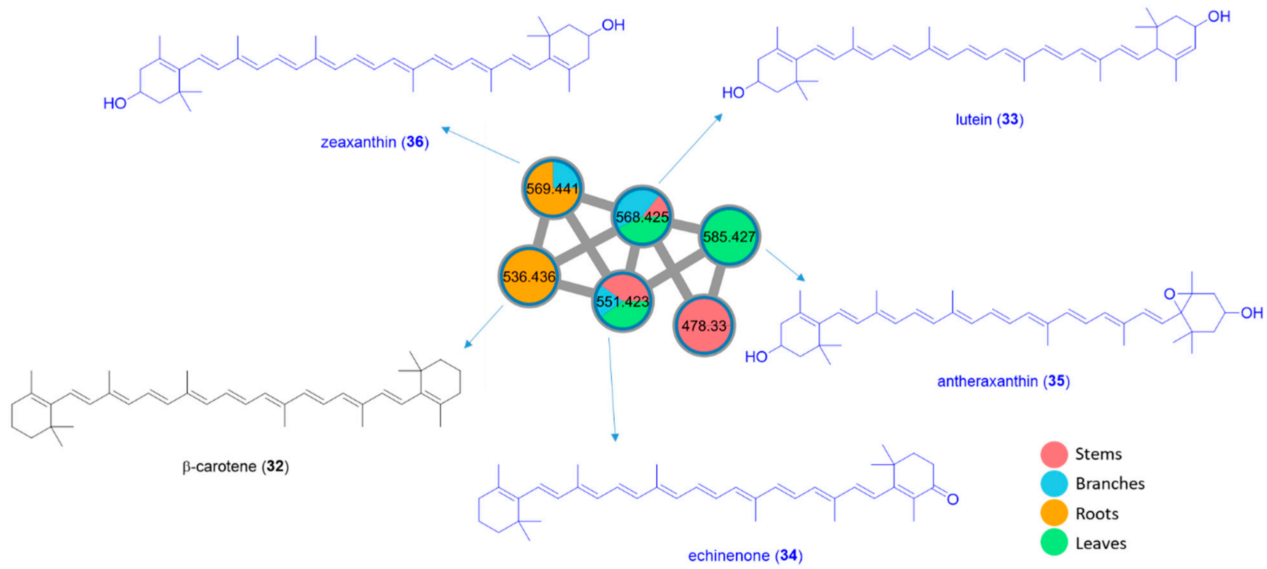

Figure S7 - Molecular family of carotenoids present in the ethanolic extracts of stems, branches, roots and leaves of *Piper rivinoides*. In blue, substances annotated through manual inspection of the data, and in black, substances annotated by comparison with the GNPS library. All substances were proposed based on fragmentations that gave rise to characteristic ions and fragmentation profiles.

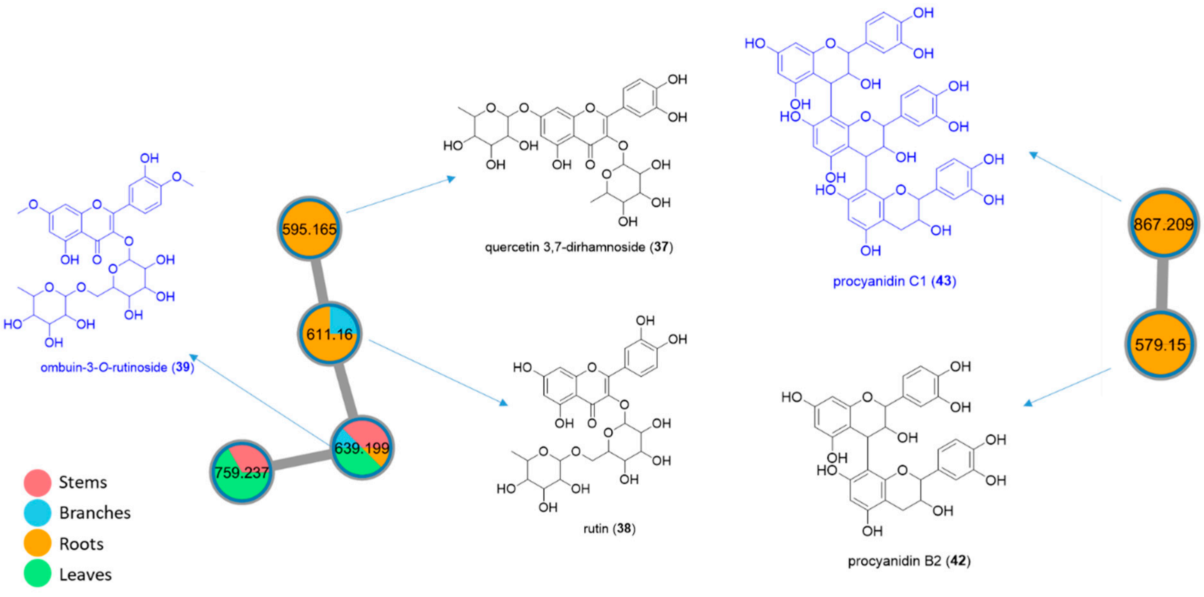

Figure S8 - Molecular families of procyanidins and glycosyl flavanoids present in the ethanolic extracts of stems, branches, roots and leaves of *Piper rivinoides*. In blue, substances annotated through manual inspection of the data, and in black, substances annotated by comparison with the GNPS library. All substances were proposed based on fragmentations that gave rise to characteristic ions and fragmentation profiles.

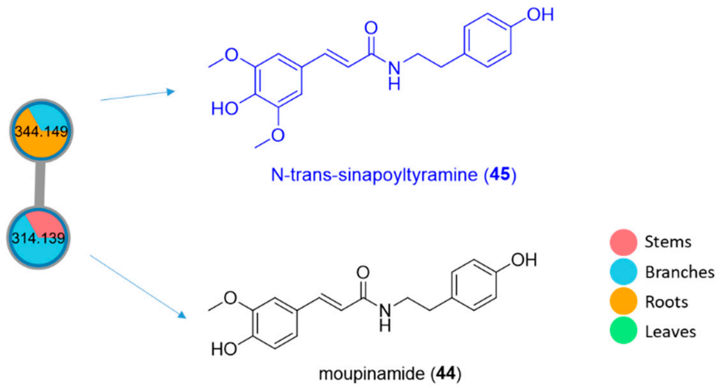

Figure S9 - Molecular family of amides present in the ethanolic extracts of stems, branches, roots and leaves of *Piper rivinoides*. In blue, substances annotated through manual inspection of the data, and in black, substances annotated by comparison with the GNPS library. All substances were proposed based on fragmentations that gave rise to characteristic ions and fragmentation profiles.

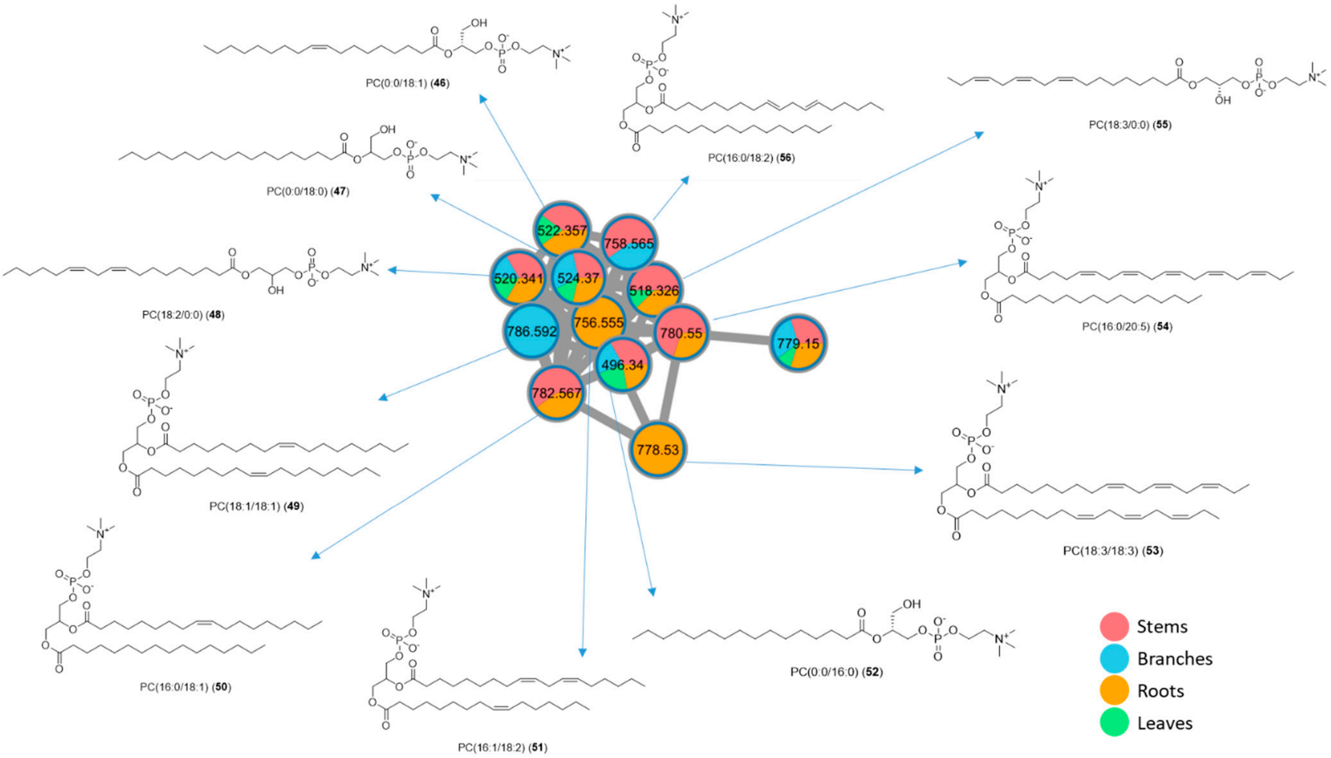

Figure S10 - Molecular family of glycerophosphocholines present in the ethanolic extracts of stems, branches, roots and leaves of *Piper rivinoides*. In black substances annotated by comparison with the GNPS library. All substances were proposed based on fragmentations that gave rise to characteristic ions and fragmentation profiles.

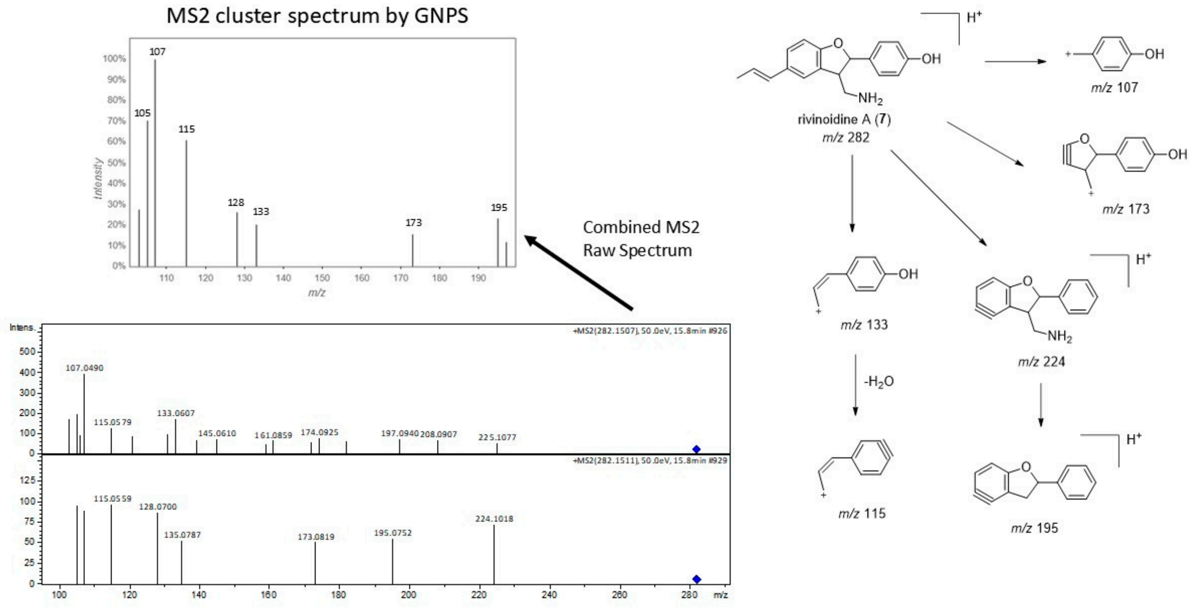

Figure S11 - Mass spectra (ESI, positive mode, MS^2) and proposed fragmentation pathway for the neolignan rivinoidine A (**7**). The MS^2 spectrum at the top of the figure represents the combined spectrum (generated by GNPS) derived from the different MS^2 detected within the retention time window corresponding to the compound (UPLC-MS/MS).

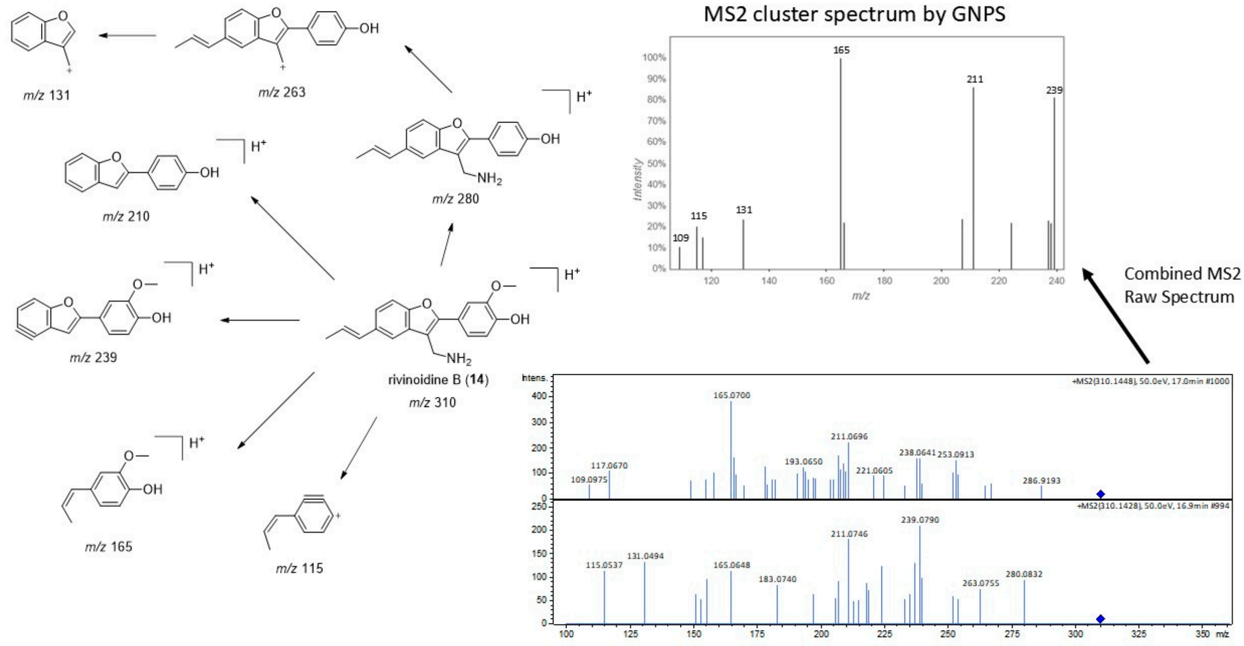

Figure S12 - Mass spectra (ESI, positive mode, MS^2) and proposed fragmentation pathway for the benzofuran-type neolignan rivinoidine B (**14**). The MS^2 spectrum at the top of the figure represents the combined spectrum (generated by GNPS) derived from the different MS^2 detected within the retention time window corresponding to the compound (UPLC-MS/MS).
